# Neutrophil-Camouflaged Stealth Liposomes for Photothermal-Induced Tumor Immunotherapy Through Intratumoral Bacterial Activation

**DOI:** 10.3390/pharmaceutics17050614

**Published:** 2025-05-05

**Authors:** Xinxin Chen, Jiang Sun, Tingxian Ye, Fanzhu Li

**Affiliations:** 1 School of Pharmaceutical Sciences, Zhejiang Chinese Medical University, Hangzhou 310053, China; m13884490537@163.com (X.C.); yetingxian123@foxmail.com (T.Y.); 2 Jinhua Academy of Zhejiang Chinese Medical University, Jinhua 321015, China; sunj500@163.com

**Keywords:** intratumor bacteria, photoimmunotherapy, neutrophil, breast cancer, tumor immune microenvironment

## Abstract

**Objective**: *F. nucleatum*, a tumor-resident bacterium colonizing breast cancer (BC), results in an immunosuppressive microenvironment and facilitates tumor growth and metastasis. This study aimed to develop a neutrophil-based liposome delivery system designed for dual-targeted elimination of tumor cells and *F. nucleatum*, while simultaneously upregulating pathogen-associated molecular patterns and damage-associated molecular patterns to potentiate tumor immunotherapy. **Methods**: The liposomes (PD/GA-LPs) loaded with the perylene diimide complex (PD) and gambogic acid (GA) were fabricated via the extrusion method. Subsequently, comprehensive evaluations including physicochemical characteristics, antibacterial activity, antitumor effect, and immunomodulatory effect evaluation were systematically conducted to validate the feasibility of this delivery system. **Results**: The resulting PD/GA-LPs exhibited a dynamic size (121.3 nm, zeta potential −44.1 mV) and a high encapsulation efficiency of approximately 78.1% (PD) and 91.8% (GA). In addition, the optimized PD/GA-LPs exhibited excellent photothermal performance and antibacterial efficacy. In vitro cellular experiments revealed that PD/GA-LPs exhibited enhanced internalization by neutrophils, followed by extracellular trap-mediated release, ultimately significantly inhibiting tumor cell proliferation and inducing immunogenic cell death. During in vivo treatment, PD/GA-LPs exhibited targeted tumor accumulation, where *F. nucleatum*-driven PD reduction activated near-infrared-responsive photothermal ablation. When combined with GA, this delivery system effectively eliminated tumor cells and *F. nucleatum*, while facilitating the subsequent T-cell infiltration. **Conclusions**: This strategy amplified the antitumor immune response, thus leading to effective treatment of BC and prevention of metastasis. In summary, this approach, grounded in the distinct microecology of tumor and normal tissues, offers novel insights into the development of precise and potent immunotherapies for BC.

## 1. Introduction

Breast cancer (BC), particularly triple-negative breast cancer, persists as one of the most threatening malignancies in women globally [1]. According to statistics, an alarming number of over 1 million new BC cases are diagnosed annually worldwide [2]. Current therapeutic strategies for BC remain hampered by the scarcity of druggable molecular targets and reliable prognostic biomarkers, frequently resulting in tumor recurrence and metastatic progression [3]. Immune checkpoint inhibitors (ICIs), particularly PD-1/PD-L1-blocking antibodies, have emerged as transformative components in the BC treatment paradigm, demonstrating clinical efficacy in KEYNOTE-355 and other trials [4,5]. Despite the demonstrated potency of PD-1/PD-L1 inhibitors, the clinical outcomes in BC patients remain suboptimal, primarily attributed to insufficient tumor-infiltrating lymphocyte density and hypoxia-induced immunosuppressive tumor immune microenvironments (TIME). Strikingly, objective response rates to these agents in BC cohorts have plateaued at approximately 4.7% across pivotal clinical trials [6,7]. This critical unmet need underscores the imperative to develop multimodal strategies that synergistically potentiate immunotherapeutic efficacy through dual modulation of immune activation and TIME remodeling.

Accumulating evidence has shown that intratumoral bacteria constitute a pivotal component of the TIME, significantly affecting the TIME and the outcomes of immunotherapy [8,9]. Intratumoral bacteria not only compromise immune surveillance by facilitating tumor immune evasion and activating immunosuppressive pathways within the TIME [10,11] but also promote the metastasis and progression of BC through the secretion of specific metabolites and the reconfiguration of the tumor cytoskeleton [12]. In BC, the intratumoral bacteria are predominantly characterized by anaerobic and facultative anaerobic species, as evidenced by 16S rRNA sequencing analyses [13]. Fusobacterium nucleatum (*F. nucleatum*), an opportunistic oral-gut pathobiot, has been implicated in the colonization of mammary tumors and the potentiation of metastasis through upregulation of tumor-derived matrix metalloproteinases [14]. Additionally, *F. nucleatum* exhibits selective adhesion to BC via Fap2 lectin-mediated binding to Gal-GalNAc disaccharide-enriched tumor surfaces [15]. This pathogen-tumor interaction triggers immune evasion through Fap2-dependent engagement of inhibitory checkpoints, while simultaneously suppressing antitumor immunity by impairing CD8^+^T cell infiltration through the CXCL10/CXCR3 axis disruption [16]. Although the role of intratumoral bacteria within the tumor microenvironment has not been fully elucidated, it is clear that their eradication offers a unique strategy for improving immunotherapy efficacy. Notably, eliminating these intratumoral bacteria could paradoxically enhance tumor immunogenicity through pathogen-associated molecular pattern (PAMP) release, thereby activating dendritic cell-mediated neoantigen presentation [17,18]. Currently, the treatment of bacterial infections primarily relies on antibiotics. However, systemic antibiotic treatment may have a detrimental effect on the efficiency of ICIs through interfering with human microecological disorder [19]. Therefore, finding an efficient local intratumoral bacterial clearance strategy is crucial for improving the efficiency of tumor treatment.

**Scheme 1:**
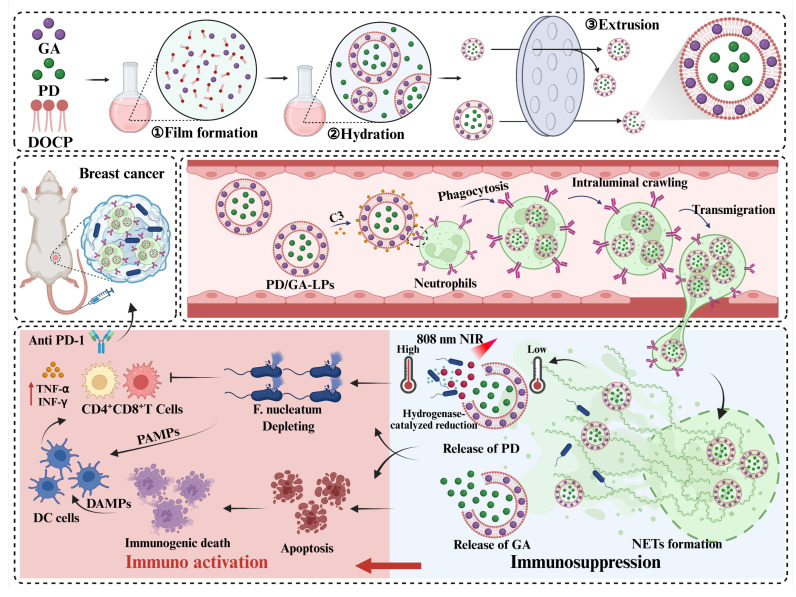
The schematic diagram of the mechanism of the PD/GA-LPs triggered by intratumoral bacteria to enhance anti-tumor immunity.

Photothermal therapy (PTT) has attracted tremendous attention for its potential in localized tumor treatment and antibacterial applications [20]. Notably, PTT-induced immunogenic cell death (ICD) can reshape immunosuppressive TIME [21]. Perylene diimide derivatives (PDD), a class of n-type organic semiconductors renowned for their exceptional fluorescence quantum yields, have emerged as potent photothermal agents for bacteria-selective photothermal ablation. This functionality arises from their electron-deficient diimide core, which is spontaneously reduced to stable radical anions under the hypoxic conditions characteristic of anaerobic or facultative anaerobic bacterial microenvironments. This enables targeted photothermal conversion via microenvironment-responsive electron transfer mechanisms [22,23]. This unique characteristic allows PDD to function as a precise photothermal agent in tumor photothermal therapy, where it exerts excellent photothermal effects at 808 nm. However, systemic administration of PDD posed multiple challenges, including poor distribution in vivo and rapid blood clearance [24]. Cell-based nanoparticle delivery systems have advanced dramatically in recent years and progressively emerged as one of the most significant ways for augmenting tumor targeting [25]. As the most abundant immune cells, neutrophils (NEs) have been widely used to carry therapeutic agents for antitumor therapy. Compared with traditional nano-delivery systems, NE-based drug delivery systems exhibit many advantages, such as high biocompatibility, inherent targeting capabilities, ability to cross biological barriers, and low in vivo immunogenicity. These advantages have injected new vitality into the tumor-targeted delivery of drugs. Once loaded with these medications, neutrophils are capable of crossing the endothelial barrier and selectively releasing the therapeutic agents in tumor tissues under the stimulation of inflammatory factors [26]. According to research, inverse phosphocholine lipids (DOCPs) rapidly enrich complement fragment iC3b, which mediates neutrophil endocytosis via complement receptor 3 phagocytosis [27]. Inspired by this, we constructed a novel neutrophil-mediated delivery system by loading PDD and gambogic acid (GA) into neutrophils (Scheme 1). This delivery system could precisely target tumor tissue, subsequently ablating tumor cells and intratumoral bacteria, releasing damage-associated molecular patterns (DAMPs), thereby enhancing the response to immunotherapy. Moreover, dead intratumoral bacteria can act as immunostimulants, thereby promoting T cell infiltration at the tumor site via the release of PAMP. Through this multiple treatment strategy, we aim to maximize the immune response against the tumor and modulate the TIME. Ultimately, this delivery strategy can inhibit tumor growth and metastasis, enabling effective tumor treatment.

## 2. Materials and Methods

### 2.1. Materials

2-((2,3-bis(oleoyloxy)propyl)dimethylammonio)ethyl hydrogen phosphate (DOCP) and cholesterol were purchased from Shanghai Advanced Vehicle Technology (AVT) Co., Ltd. (Shanghai, China); Perylene diimide derivatives was obtained from Shanghai Medicilon Inc (Shanghai, China); Gambogic acid (GA), HEPES buffer, lipopolysaccharide, DAPI and indocyanine green (ICG) were provided by Shanghai Aladdin Biochemical Technology Co., Ltd. (Shanghai, China); FITC-conjugated anti-mouse Ly-6G/Ly-6C, PE-conjugated anti-mouse CD11b, FITC Plus anti-mouse CD3, PE anti-mouse CD4 and APC anti-mouse CD8 were obtained from Wuhan Sanying Biotechnology Co., Ltd. (Wuhan, China); The anti-HSP90 antibodies were supplied from Abcam Shanghai Trading Co., Ltd. (Shanghai, China); Mouse bone marrow neutrophil isolation kit and mouse peripheral blood lymphocyte separation kit were purchased from Beijing Solebao Technology Co., Ltd. (Beijing, China). ELISA kits were supplied by Jiangsu enzyme immunoassay Industry Co., Ltd. (Nanjing, China). 

### 2.2. Bacteria, Cell Lines, and Animals

Fusobacterium nucleatum (*F. nucleatum*) ATCC 10953 was purchased from Guangdong Microbial Culture Collection Center (Guangzhou, China) and was grown anaerobically on brain heart infusion (BHI) agar plates supplemented with 5% defibrinated sheep blood. 4T1 cells were provided by the Cell Bank of the Chinese Academy of Sciences (Shanghai, China). 4T1 cells were cultured in RPMI 1640 medium containing 10% FBS and penicillin/streptomycin (1%, *w*/*v*). Balb/c mice (female, 6–8 weeks, 20–30 g) were supplied by Shanghai Slack Laboratory Animal Co., Ltd. (Shanghai, China) and fed in an SPF laboratory. All animal experiments were conducted following the guidelines of the Institutional Animal Care Committee of Zhejiang Chinese Medical University.

### 2.3. Preparation of PD/GA-LPs

The perylene diimide complex (PD) was synthesized through host–guest complexation between PDD and cucurbit[7]uril (CB[7]) to suppress the dimerization and quenching of PD radical anions (Figure S1) [22,23]. The PD/GA co-loaded liposomes (PD/GA-LPs) were prepared using a sequential thin-film hydration and extrusion method [28]. Briefly, DOCP, cholesterol, and GA (10:3:1 molar ratio) were dissolved in 1 mL of chloroform and rotary evaporated at 25 °C to form a homogeneous lipid film. The films were subsequently hydrated with 0.5 mL of HEPES buffer solution (10 mM HEPES, 100 mM NaCl, pH 7.4) containing PD at graded concentrations (50–500 μg/mL) for 30 min at 37 °C. The resulting cloudy suspension was extruded 21 times through 200 nm and 100 nm polycarbonate membranes using an extruder (HOMOEX-25, HOMOEX, Shanghai, China) to achieve uniform unilamellar vesicles. Final formulations were purified on a Sephadex G-25 PD-10 column and then stored at 4 °C until used. The PD-loaded liposomes (PD-LPs), GA-loaded liposomes (GA-LPs), and DiI-labeled liposomes (DiI-LPs) were also suitable for the above-mentioned method under dark conditions.

### 2.4. Characterization of PD/GA-LPs

Particle size distribution and zeta potential, as well as the polydispersity index (PDI) of liposomal formulations, were determined by dynamic light scattering (DLS) and laser doppler electrophoresis system (Nano-ZS90, Malvern, Worcestershire, UK). The morphological evaluation of PD/GA-LPs was performed using a transmission electron microscope (TEM, H-7650, Hitachi, Japan). For TEM imaging, samples were prepared by negatively staining them with 2.0% phosphotungstic acid hydrate for 3 min, followed by 5 min of infrared drying on 200-mesh copper grids. The encapsulation efficiency (EE) and drug loading (DL) of PD and GA were quantified using high-performance liquid chromatography (HPLC, Waters, Massachusetts, USA) at 370 nm and 500 nm, respectively, with a flow rate of 1 mL/min at a column temperature of 25 °C. The UV–vis absorption spectra of PD/GA-LPs were monitored using a multifunctional microplate reader (SpectraMax M2, Molecular Devices, Silicon Valley, CA, USA).

### 2.5. In Vitro Drug Release

The in vitro release profiles of PD and GA that form PD/GA-LPs were evaluated using a dialysis method in a phosphate buffer solution (PBS, pH 7.4) containing 20% ethanol, maintaining sink conditions. Briefly, 5 mL of PD/GA-LPs (equivalent to 0.38 mg GA and 2.02 mg PD) were loaded into dialysis bags (MWCO 3500 Da) and immersed in 50 mL of release medium under continuous agitation (37 °C, 150 rpm). At predetermined time intervals, 1 mL of the dialysis solution was taken out and replaced with an equal volume of fresh pre-warmed medium. The PD and GA content in each sample was examined by the HPLC method.

### 2.6. Short-Term Stability Assay

For the assessment of PD/GA-LPs stability, liposomes at a concentration of 2 mg/mL were dispersed in PBS and incubated at 37 °C for 9 days. The change in particle size and zeta-potential with respect to time was measured by a Malvern laser particle size analyzer (Nano-ZS90, Worcestershire, UK) within a predetermined time window.

### 2.7. In Vitro Bacteria-Responsive Photothermal Effect

*F. nucleatum* suspension was adjusted to 5 × 10^5^ CFU/mL in PBS and co-incubated with PD/GA-LPs for 8 h at 37 °C. Recordings of the UV–vis absorption spectra (400–900 nm) of PD/GA-LPs were acquired using a multifunctional microplate reader before and after *F. nucleatum* exposure. To evaluate the photothermal performance of PD/GA-LPs responsive to *F. nucleatum*, 2 mL of PBS, PD/GA-LPs, and a mixture of PD/GA-LPs and *F. nucleatum* with a determined concentration of PD were, respectively, irradiated with an 808 nm laser (2W/cm^2^) for 10 min. An infrared thermal imaging camera (Fotric 227S, Dallas, USA) was used to simultaneously monitor and measure thermal images at various periods. The photothermal stability of the PD/GA-LPs was evaluated by subjecting the sample to cyclic irradiation with an 808 nm NIR laser for 5 min (laser ON), followed by a 10-min period of natural cooling without NIR laser exposure (laser OFF). This process was repeated five times. Infrared thermal imaging captures of the irradiated samples were acquired to characterize their stability throughout the cycles.

### 2.8. In Vitro Antibacterial Activity

The in vitro antibacterial efficacy of PD/GA-LPs against *F. nucleatum* was evaluated using a standardized microbroth dilution assay [29]. Briefly, *F. nucleatum* suspensions were diluted to a concentration of 1 × 10^7^ CFU/mL in BHI medium. Then, 50 μL of the bacterial suspension was added to a sterile 96-well plate, followed by the addition of 100 µL of various formulations (PBS, PD-LPs, GA-LPs, and PD/GA-LPs). The concentrations of PD and GA were adjusted to 90.2 μg/mL and 3.9 μg/mL, respectively. Following 10 min of NIR irradiation (808 nm laser, 2.0 W/cm²) for designated groups (PBS + NIR, PD-LPs + NIR, PD/GA-LPs + NIR), all samples were incubated at 37 °C for 3 h with orbital shaking (150 rpm). An aliquot of a 20 μL sample was spread on BHI plates after 1000 × dilution and cultured overnight. Finally, each agar plate was photographed using a gel imaging system (GenoSens1850, Jinan, China), and the bacterial colonies were manually monitored.

### 2.9. Internalization of PD/GA-LPs into NEs

In vitro analysis of PD/GA-LP internalization by NEs was conducted using confocal laser scanning microscopy (CLSM) and flow cytometry. Briefly, bone marrow NEs were isolated from C57BL/6 mice using a mouse neutrophil isolation kit according to the manufacturer’s protocol [30]. Freshly harvested NEs (5 × 10^5^ cells/well) were seeded into glass-bottomed confocal dishes and incubated for 1 h at 37 °C and 5% CO_2_. Cells were subsequently incubated with DiI-LPs (10 μg/mL) at 37 °C in complete darkness for 4 h. Following three washes with ice-cold PBS, NEs were stained with FITC-conjugated anti-mouse Ly-6G/Ly-6C (1 μL, 500 μg/mL) for surface marker identification and DAPI for nuclear counterstaining. Finally, NEs were fixed with 4% paraformaldehyde and examined using a CLSM to detect the internalization of DiI-LPs by NEs.

To further validate DiI-LP internalization by NEs, purified NEs were seeded in 6-well plates (5 × 10^5^ cells/well) and incubated with DiI-LPs (DiI: 5 μg/mL) in a humidified incubator (5% CO_2_, 37 °C) for 4 h. After surface staining with FITC-conjugated anti-mouse Ly-6G/Ly-6C and PBS resuspension, cellular fluorescence was quantified using a CytoFLEX flow cytometer (Beckman Coulter, Fullerton, CA, USA). The instrument was equipped with a 488 nm laser for FITC and a 561 nm laser for DiI, and 10,000 events per sample were collected, gated on the Ly-6G⁺ population.

### 2.10. Cytotoxicity of PD/GA-LPs Toward NEs

The in vitro cytotoxicity of PD/GA-LPs against NEs was evaluated using the MTT assay. Initially, NEs were seeded in a 96-well plate at a density of 1 × 10^4^ cells/well and pre-equilibrated in RPMI 1640 medium for 1 h. Following that, cells were treated with PD/GA-LPs (equivalent GA ranging from 0.1 μg/mL to 10 μg/mL) for 12 h, followed by incubation with MTT (0.5 mg/mL final concentration) for 4 h. Subsequently, formazan crystals were solubilized with DMSO (150 μL per well), and the absorbance was measured at 570 nm in a multifunctional microplate reader (SpectraMax M2, Molecular Devices, CA, USA).

The in vitro activity of PD/GA-LPs on NEs was assessed using a Senescence β-Galactosidase Staining Kit. In brief, NEs were first pre-cultured in RPMI 1640 medium for 1 h after being seeded in a 6-well plate at a density of 5 × 10^5^ cells per well. Subsequently, fresh medium containing the corresponding PD/GA-LPs was added to each well for further incubation. After 12 h of incubation, NEs were stained with the Senescence β-Galactosidase Staining Kit (Beyotime, Shanghai, China) following the manufacturer’s instructions and observed using an optical microscope. Diff-Quik staining and DAPI staining were used to evaluate the changes in NEs morphology before and after cellular internalization of PD/GA-LPs.

### 2.11. Endothelial Permeability

The migratory capacity of PD/GA-LPs-loaded NEs was assessed utilizing a 24-well Transwell migration assay (3 μm pores, Corning Costar). First, purified NEs were exposed to the culture medium (referred to as blank NEs group) or PD/GA-LPs (referred to as PD/GA-LPs-NEs group) for 0.5 h at 37 °C in 5% CO_2_. Following PBS washing, the NEs were collected and planted in the apical chamber at a density of 2 × 10^5^ cells per well, while the lower chambers contained 600 μL of culture medium with 0 or 10 nM fMLP. Following a 30-minute incubation, NEs that migrated to the lower membrane surface were fixed with 4% paraformaldehyde, stained with 0.1% crystal violet for 30 min, and captured with an inverted fluorescence microscope (Nikon Ti-S, Tokyo, Japan).

### 2.12. PD/GA-LP Release from PD/GA-LPs-NEs

The release behavior of PD and GA from PD/GA-LPs-NEs was investigated by determining the amount of PD and GA in the supernatant following co-culture with phorbol 12-myristate 13-acetate (PMA) [31]. The PD/GA-LPs-NE suspension was seeded on the lower chamber of the Transwell plates (3 μm pores, Corning Costar) at a density of 2 × 10^5^ cells per well and pre-cultured in a 37 °C incubator for 1 h. The upper chamber was then filled with fresh culture medium (0.5 mL) containing PMA at a concentration of 50 nM and incubated for predetermined intervals. An aliquot of 1 mL of the samples was withdrawn from the lower chamber and centrifuged at 400 g for 5 min. The supernatant was disrupted using an ultrasonic homogenizer and quantified using the HPLC method.

To identify whether the drug release from NEs was mediated by PMA-induced formation of neutrophil extracellular traps (NETs), PD/GA-LPs-NEs (2 × 10^5^ cells/well) were seeded, treated with PMA, and stained for 20 min with FITC-conjugated anti-mouse Ly-6G/Ly-6C and DAPI, respectively. CLSM was used for imaging. In addition, the co-cultured NEs were extracted by centrifugation (500 g for 10 min at 4 °C), immersed in 2.0% glutaraldehyde overnight, and photographed using scanning electron microscopy (SEM).

To determine whether DiI-LPs released from NEs could be taken up by tumor cells, 4T1 cells were co-cultured with DiI-LPs-NEs (DiI: 5 μg/mL) in 6-well plates and grown for 4 h at 37 °C with 5% CO_2_. The cells were then stained with 1 μg/mL DAPI for 10 min and subsequently observed under a CLSM.

### 2.13. Cytotoxicity and Cell Apoptosis Induced by PD/GA-LPs-NEs

The MTT assay was utilized to visually assess the cytotoxicity of PD/GA-LPs-NEs against 4T1 cells. 4T1 cells (1 × 10^4^ cells per well) were seeded in 96-well plates and cultured for 24 h. Cells were then exposed to various formulations at different dilutions with or without 808 nm laser irradiation. PD/GA-LPs-NEs were pretreated for 8 h with *F. nucleatum* before the addition of various formulations to the cultures. Then, both the untreated and *F. nucleatum*-treated PD/GA-LPs-NEs were centrifuged at 500 g for 5 min, and the obtained supernatants were cultivated with 4T1 cells for 24 h. At the end of the incubation period, the viability of 4T1 cells was determined using the MTT method.

The apoptosis induced by PD/GA-LPs-NEs in 4T1 cells was detected using an Annexin V-FITC/PI apoptosis detection kit (Beyotime, Shanghai, China). Briefly, 4T1 cells were seeded in a 6-well plate and incubated overnight, followed by treatment with different formulations for 3 h. The cells were then irradiated with an 808 nm laser (2 W/cm^2^, 5min) and incubated for another 1 h. Finally, the cells were trypsinized, harvested in a 0.5 mL flow tube, and stained with Annexin V-FITC and PI, respectively. The percentage of apoptosis was determined by flow cytometry.

To further investigate the cytotoxicity of PD/GA-LPs-NEs, live/dead cell staining was performed. After various treatments, the cells were detected with a Calcein-AM/PI kit (Beyotime, Shanghai, China) and directly observed using a CLSM.

### 2.14. Intracellular ROS Detection

For ROS detection, 4T1 cells were seeded into 6-well plates and incubated overnight at 37 °C. The cells were then exposed to PBS (with or without laser), GA-LPs (2 μg/mL), and *F. nucleatum*-pretreated PD/GA-LPs with an equal concentration of PD (90 μg/mL) and GA concentration (2 μg/mL) for 3 h. After treatment, the cells were exposed to an 808 nm laser (2 W/cm^2^, 5 min) and incubated for 1 h. Finally, cells from each group were stained with DCFH-DA (10 μM) for 30 min and finally observed under a CLSM.

### 2.15. Regulation of HSP90 and HSP70 with PD/GA-LPs-NEs In Vitro

To assess the ability of PD/GA-LPs-NEs to suppress HSP90/HSP70 in vitro, 4T1 cells were seeded into 6-well plates and incubated for 24 h. Cells were then treated with various formulations (PBS, PD-LPS-NEs + NIR, free GA, PD/GA-LPs-Nes, and PD/GA-LPs-NEs + NIR) for 24 h. After treatment, the cells were fixed with 4% (*v*/*v*) formaldehyde for 15 min and stained with anti-mouse HSP90 antibody or anti-mouse HSP70 antibody. Subsequently, the cells were incubated with Alexa Fluor 488 and 594 goat anti-rabbit IgG for 2 h. Finally, cells were labeled with DAPI for 10 min and visualized using a CLSM.

### 2.16. Tumor Penetration and Cytotoxicity in 4T1 Tumor Spheroids

Tumor spheroids of 4T1 cells were prepared by the low-adhesion culture method as previously described [32,33]. Briefly, 4T1 cells (1 × 10^4^ cells/well) were seeded in a 96-well round-bottom plate (ultra-low attachment, Corning) and cultured in RPMI 1640 medium for 4 days. *F. nucleatum* was then inoculated into the 96-well plate containing mature tumor spheroids and returned to the incubator. After 2 h of infection, the tumor spheroids were thoroughly washed with PBS and then incubated in the culture medium containing gentamicin (2.5 μg/mL) for an additional 16 h. During this period, spheroid growth was monitored via light microscopy, and uniform-sized spheroids were selected for subsequent experiments. The resulting tumor spheroids were incubated with free ICG, ICG-LPs, or ICG-LPs-NEs at an ICG concentration of 20 μg/mL for 4 h. After washing and fixing with paraformaldehyde, tumor spheroids were imaged using a CLSM.

To assess the cytotoxicity of PD/GA-LPs-NEs against tumor spheroids, spheroid size was measured directly. After treatment with PBS, PD/GA-LPs, or PD/GA-LPs-NEs for 4 h, 4T1 tumor spheroids were either exposed to an 808 laser (2 W/cm^2^, 5 min) or left untreated. At specific time intervals, tumor spheroids were rinsed three times with PBS and observed under a light microscope.

### 2.17. Analysis of Immunogenic Cell Death

To directly visualize photothermal-induced ICD, 4T1 cells were incubated with PBS, PD-LPs-NEs, GA-LPs-Nes, and PD/GA-LPs-NEs. After 4 h of incubation, the cells were irradiated with an 808 nm laser (2 W/cm^2^) for 5 min and incubated for another 8 h. Subsequently, the cells were washed with cold PBS, stained with primary anti-HMGB1 antibody or anti-CRT antibody, and incubated overnight at 4 °C. Following this, Alexa Fluor 488 and 594 goat anti-rabbit IgG were used to incubate the above cells. Finally, the cells were stained with DAPI and imaged directly using a CLSM. Additionally, the intracellular ATP level of cells was detected by an ATP assay kit.

### 2.18. In Vivo Fluorescence Imaging and Biodistribution

To assess the intratumoral accumulation and biodistribution of PD/GA-LPs, a bacterial-colonized tumor model was established by intratumoral injection of *F. nucleatum* suspension (5 × 10^6^ CFU/mL, 100 μL) into the tumor site when the tumor volume reached 80 mm³ [17,34]. 4T1 tumor-bearing mice were randomly divided into two groups and intravenously administered free ICG and ICG-LPs at an equivalent ICG dose of 1 mg/kg. In vivo fluorescence imaging (Ex = 710 nm, Em = 745 nm) was performed using a small animal optical imaging system (Perkin Elmer, Waltham, MA, USA) at predetermined time intervals (1, 6, 12, and 24 h post-injection). For ex vivo fluorescence imaging, the mice were euthanized at 24 h post-injection and major organs (heart, liver, spleen, lung, kidney, and tumor) were harvested to further explore the biodistribution of PD/GA-LPs.

### 2.19. In Vivo Photothermal Effect

*F. nucleatum*-colonized orthotopic 4T1 tumor-bearing mice were randomly divided into two groups (n = 3). PD/GA-LPs dispersed in PBS were intravenously injected into the tumor-bearing mice. After 12 h, the mice were irradiated by an 808 nm laser (2 W/cm^2^, 5 min). The temperature changes at the tumor site were monitored and recorded using an infrared thermal imaging camera at specific time points.

### 2.20. In Vivo Antitumor Evaluation on a Bacteria-Colonized Tumor Model

4T1 tumor-bearing mice were randomly allocated into 7 groups (n = 5), including PBS group, PD + NIR group, αPD-1 group, free GA group, PD/GA-LPs group, PD/GA-LPs + NIR group, and PD/GA-LPs + NIR + αPD-1 group. Mice in each group were intravenously injected with 200 μL of the corresponding formulations containing equivalent PD (300 μg/mL) and GA (50 μg/mL) concentrations. At 12 h post-injection, tumors in each group were subjected to an 808 nm laser irradiation (2 W/cm^2^) for 5 min, followed by tail vein injection of αPD-1 (1 mg/mL). All mice received intravenous injections of the formulations via the tail vein once every two days for a total of three doses. During the treatment period, tumor volumes were recorded every other day. After 15 days of treatment, the mice were euthanized, and tumors were harvested. A portion of the tumor was homogenized, diluted, and inoculated onto Columbia CNA blood agar plates. Another portion was rinsed with saline, fixed in 4% formaldehyde, and analyzed by HE staining. Ki67 and TUNEL staining were performed to assess proliferation inhibition and apoptosis induction. The FITC Plus anti-mouse CD3, PE anti-mouse CD4, and APC anti-mouse CD8 were employed to detect tumor-infiltrating lymphocyte content within tumor tissues using flow cytometry. The plasma levels of IL-2, IL-1β, IL-6, IL-10, IFN-γ, and TNF-α were determined using ELISA kits.

### 2.21. Immune Memory Effect

Following therapeutic interventions in 4T1 tumor-bearing mice, the primary tumors were surgically resected on day 20 post-treatment. After a 7-day recovery period, a metastatic challenge was performed by intravenous injection of 4T1 cells (5 × 10^5^ cells per mouse) via the tail vein. Pulmonary metastases were quantified through macroscopic imaging and histopathological analysis (4% paraformaldehyde-fixed, H&E-stained sections) at 14 days post-inoculation.

### 2.22. Biosafety Evaluation

The blood compatibility of PD/GA-LPs was assessed using fresh murine erythrocytes. Initially, a 2% erythrocyte suspension was prepared by suspending 0.5 mL of red blood cells in 24.5 mL of normal saline. Subsequently, 0.5 mL of the suspension was incubated with PD/GA-LPs at lipid concentrations of 50–800 μg/mL at 37 °C for 1 h. Physiological saline and deionized water served as the negative and positive controls, respectively. After centrifugation (1500 rpm, 10 min), the supernatants were collected and hemoglobin release was measured at 570 nm using a multifunctional microplate reader. Each analysis was repeated three times.

For in vivo biosafety evaluation, the major organs (heart, liver, spleen, lung, and kidney) were harvested, fixed in 4% paraformaldehyde, and subjected to H&E staining to assess histopathological changes. The whole blood samples were collected for a standard blood test and biochemistry assay. The skin damage was evaluated using a ‘one-facula’ experiment [35]. Temperature changes at the tumor sites and adjacent skin under the same spot were monitored by a thermal imager. At the experimental endpoint, tumor and adjacent skin tissues were collected for H&E staining to evaluate tissue damage.

### 2.23. Statistical Analysis

The statistical significance was evaluated through a Students’s *t*-test and one-way ANOVA followed by a post hoc Tukey test using IBM SPSS Statistics 22. All data were performed in triplicate, representing their results as mean ± standard deviation (SD) unless stated otherwise. The values reported with *p* < 0.05 and below were considered statistically significant. All graphs were plotted using GraphPad Prism 7 and Adobe Illustrator 2022.

## 3. Results and Discussion

### 3.1. Preparation and Characterization of PD/GA-LPs

**Figure 1:**
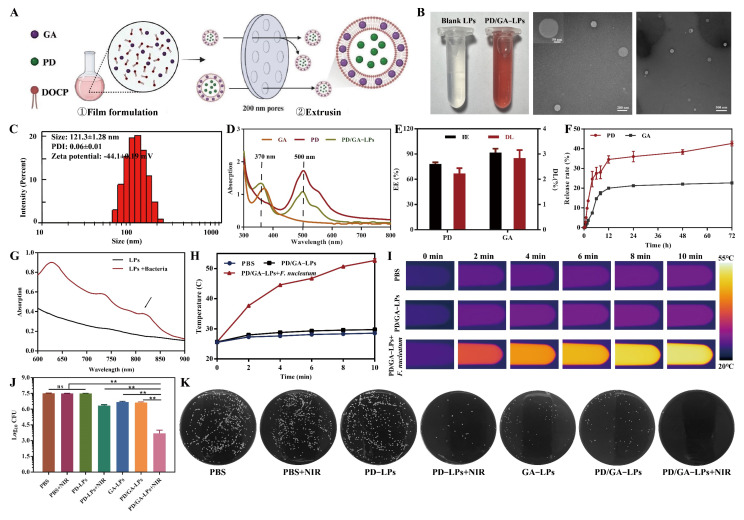
Preparation and characterization of PD/GA-LPs. (**A**) Schematic illustration of PD/GA-LP preparation. (**B**) The representative photograph and TEM images of PD/GA–LPs (Scale bar: 200 μm, 500 μm). (**C**) Hydrodynamic size distributions of PD/GA-LPs. (**D**) UV–vis spectra of free GA, free PD, and PD/GA-LPs. (**E**) The DL and EE of PD/GA-LPs (n = 3). (**F**) The cumulative GA and PD release from PD/GA-LPs (n = 3). (**G**) UV–vis absorption spectra of PD/GA-LPs before and after incubation of *F. nucleatum*. (**H**) Infrared thermographic maps of PD/GA-LPs in the presence of *F. nucleatum* under NIR irradiation (808 nm, 2 W/cm^2^) (n = 3). (**I**) Temperature profile of PD/GA–LPs with 808 nm laser irradiation for different durations. (**J**) Colony-forming unit (CFU) ratio of *F. nucleatum* with various formulations under 808 nm NIR irradiation for 10 min (n = 3). (**K**) Colony plate images of *F. nucleatum* treated with various formulations (ns indicates *p* > 0.05; ** *p* < 0.01).

UV–vis and fluorescence spectroscopy revealed that the optimal supramolecular complexation between PDD and CB[7] occurred at a 1:2 molar ratio in aqueous solution (Figure S2). PD/GA-LPs were prepared using an enhanced thin-film dispersion method (Figure 1A). The resulting PD/GA-LPs formed ruby-red aqueous solutions, and TEM images confirmed their spherical shape with a diameter of 110–130 nm (Figure 1B). DLS analysis further characterized the liposomes, showing an average hydrodynamic size of 121.3 nm and a zeta potential of −44.1 mV, consistent with TEM data (Figure 1C). These results endowed PD/GA-LPs with high stability, ultra-long cycling stability, and NE-targeting potential. This observation aligned with previous reports by Chen and Mathur, who demonstrated that liposomes with a hydrodynamic diameter of ~200 nm and a zeta potential of −20 mV displayed enhanced stability and specific targeting toward NEs [36,37]. UV–vis spectrometry revealed that PD/GA-LPs retained the characteristic absorption peaks of GA (370 nm) and PD (500 nm) (Figure 1D). The EE of PD and GA in PD/GA-LPs was 78.1% and 91.8%, respectively, with corresponding DL rates of 2.45% and 0.97% (Figure 1E). In vitro drug release behavior of PD/GA-LPs was evaluated in PBS (pH 7.4). The results indicated that the release of PD and GA from PD/GA-LPs exhibited a time-dependent behavior, with an initial burst release within 12 h followed by a sustained release within 60 h. More than 40% of PD was released from PD/GA-LPs within 72 h, while less than 25% of GA was released up to 72h (Figure 1F). Short-term stability studies showed no significant particle size increase or aggregation after 9 days of incubation at 37 °C (Figure S3), confirming the excellent colloidal stability of PD/GA-LPs under physiological conditions.

### 3.2. In Vitro Photothermal Properties and Antibacterial Activity

As previously reported, PD could generate radical anions through reduction reactions, exhibiting a strong near-infrared light absorption property [22,38]. For this purpose, UV–visible spectroscopy was employed to monitor the spectral changes before and after incubation with *F. nucleatum*. A gradual color transition from red to deep purple was observed during incubation (Figure S4), indicative of radical anion formation. UV spectral analysis further provided evidence for radical anion production. The characteristic absorption bands of PD at 500 nm and 550 nm weaken in intensity, while two new bands appeared at 728 nm and 814 nm post-incubation with *F. nucleatum* (Figure 1G and Figure S5). Consistent with previous reports, these results demonstrated that *F. nucleatum* could reduce PD to free radical anions through hydrogenase-catalyzed reduction on its surface, providing a basis for the subsequent photothermal treatment of tumors [23,38].

The in vitro photothermal properties of PD/GA-LPs were evaluated under 808 nm laser irradiation (2 W/cm^2^). As illustrated in Figure 1H,I, the temperature of PD/GA-LPs incubated with *F. nucleatum* rapidly increased from 25 °C to 52 °C, significantly exceeding the temperature rises in PD/GA-LPs alone (4.3 °C) and PBS (3.0 °C). Additionally, PD/GA-LPs maintained stable photothermal performance over five laser irradiation-cooling cycles (Figure S6), confirming their robust bacterial-triggered photothermal activity and thermal stability. In vitro antibacterial assays (Figure 1J,K) revealed that PD/GA-LPs combined with NIR irradiation exhibited superior bactericidal effects compared to controls. This synergistic effect achieved near-complete bacterial eradication through laser-induced hyperthermia, highlighting their therapeutic potential.

### 3.3. NE Loading of PD/GA-LPs and the Release Efficiency

**Figure 2:**
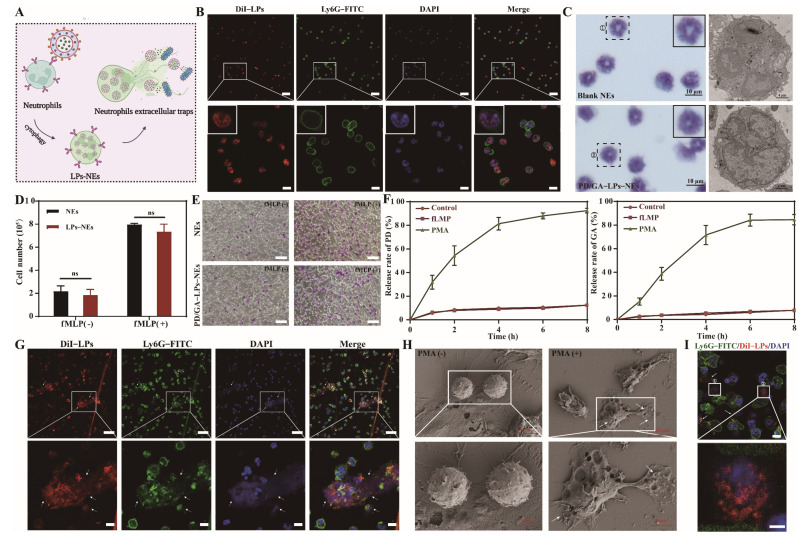
Evaluation of the biological function of PD/GA–LPs–NEs. (**A**) Schematic illustration of PD/GA–LPs–NEs formation and release in vitro. (**B**) CLSM images of NEs after co-incubation with DiI–LPs for 4 h (Scale bar: 50 μm, 10 μm). (**C**) Morphological images of NEs and PD/GA–LPsc–NEs stained with Diff-Quick, SA–β–Gal, and DAPI. (**D**) Cell numbers of NEs and PD/GA–LPs–NEs after incubation with 10 nM fMLP for 30 min (n = 3). (**E**) The chemotaxis of NEs before and after loading with PD/GA–LPs (Scale bar: 50 μm). (**F**) The amount of PD and GA released from PD/GA–LPs–NEs with varied treatments (n = 3). (**G**) CLSM images of DiI-LPs releasing from DiI–LPs–NEs (Scale bar: 50 μm). (**H**) SEM images of NETs before and after PMA incubation (Scale bar: 10 μm, 5 μm). (**I**) CLSM images of DiI–LP uptake by 4T1 cells (Scale bar: 10 μm, 5 μm) (ns indicates *p* > 0.05).

Precise delivery of nanocarriers via NEs was a crucial requirement for efficient tumor treatment [39,40]. PD/GA-LPs were phagocytosed by NEs, forming PD/GA-LPs-loaded Nes (PD/GA-LPs-NEs), which subsequently migrated to tumor sites and released their cargo via NETs. During this process, the PD/GA-LPs encountering hydrogenase produced by *F. nucleatum* caused the PD to form anionic free radicals, which exhibited photothermal effects (Figure 2A). To validate this mechanism, mature NEs were isolated and purified from mouse bone marrow. The average yield of NEs was approximately 2 × 10^6^ cells/mouse, and the percentage purity of NEs was measured to be 86.9% (Figure S7). Cellular internalization of DiI-LPs by NEs was investigated using a CLSM and flow cytometry. CLSM images revealed intense red fluorescence within NEs after 4 h of incubation (Figure 2B), demonstrating rapid nanoparticle uptake. Flow cytometry analysis further quantified the internalization efficiency of 68% for PD/GA-LPs, underscoring the high capacity of NEs to engulf nanocarriers (Figure S8). In vivo colocalization analysis demonstrated significant red fluorescence (DiI-LPs) within NEs, confirming the precise NE-targeting capability of the liposomal system (Figure S9).

To validate the efficacy of NE-mediated delivery of PD/GA-LPs, we assessed potential alterations in NEs’ physiological and biological functions. No significant differences were observed in cell morphology, nuclear integrity, or senescence status between PD/GA-LPs-NEs and blank NEs (Figure 2C and Figure S10). PD/GA-LPs loading also had no significant effect on the cellular viability of NEs (Figure S11). In vitro migration capability of PD/GA-LPs-NEs was assessed using a Transwell assay. The data in Figure 2D,E and Figure S12 indicated that PD/GA-LPs-NEs responded favorably to fMLP and accumulated rapidly in the lower chamber, which was in accordance with previous research [31,41]. As expected, the loading of PD/GA-LPs did not alter the migration ability of NEs, which was not significantly different from that of blank NEs. These data demonstrated that PD/GA-LPs-NEs preserved the physiological function of NEs and allowed for tumor site-selective drug delivery.

During tumor progression, NEs were recruited by the tumor microenvironment to inflammatory sites, where they regulated tumor growth and progression via NET formation. Capitalizing on the spatiotemporal control of NET formation, researchers have engineered neutrophil-based drug delivery systems for site-specific drug release within tumors [25,26]. To validate this approach, drug release kinetics from PD/GA-LPs-NEs were investigated. Upon stimulation with phorbol myristate acetate (PMA), 76–92% of PD and GA were released from PD/GA-LPs-NEs within 24 h, demonstrating triggered release capability under inflammatory conditions (Figure 2F). The released PD/GA-LPs maintained intact liposomal structures (Figure S13). To elucidate the release mechanism, PD/GA-LPs-NEs were co-incubated with PMA for 2 h. CLSM images revealed red fluorescent signals (DiI) that were colocalized with DAPI-stained NETs (Figure 2G), while SEM images visualized NET-mediated liposome release (Figure 2H). These data suggest that NETs formation drives rapid PD/GA-LPs release.

It was suggested that DiI-LPs-NEs might rapidly release DiI-LPs via NET formulation. Moreover, cellular uptake of DiI-LPs by 4T1 cells was investigated. In Figure 2I, arrows indicate the NEs (yellow) and 4T1 cells (white). As expected, obvious red fluorescence of DiI-LPs was observed in 4T1 cells after incubation with DiI-LPs-NEs for 4 h. Taken together, these findings indicated that PD/GA-LPs taken up by NEs could efficiently transport to tumors and then be released from NEs with the formulation of NETs for subsequent tumor treatment.

### 3.4. Cellular Cytotoxicity In Vitro

**Figure 3:**
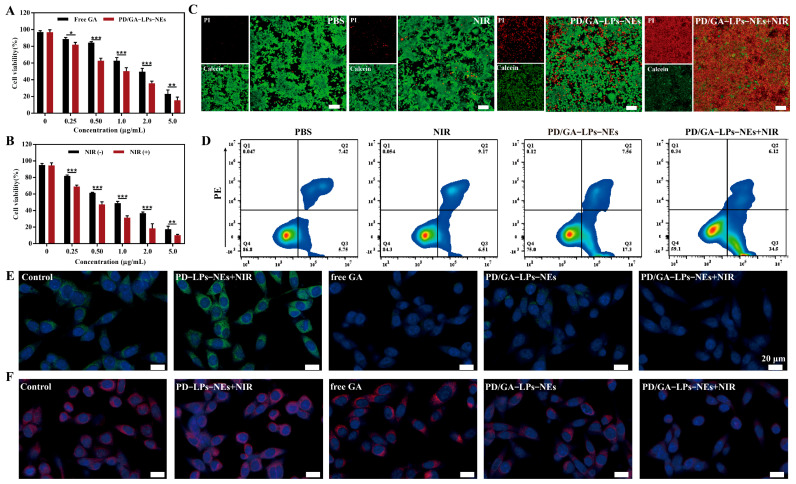
In vitro anticancer effects of PD/GA-LPs against 4T1 cells. (**A**) Cell viability of 4T1 cells incubated with various formulations for 24 h (n = 4). (**B**) Cell viability of 4T1 cells after PD/GA-LPs-NEs treatment for 24 h with or without laser irradiation (n = 4). (**C**) Live-dead staining of 4T1 treated with PBS and different formulations for 4 h (Scale bar: 100 μm). (**D**) Apoptosis analysis via an Annexin V-FITC/PI assay of 4T1 cells incubated with PBS and different formulations for 4 h (n = 3). (**E**) CLSM images of the expression of the HSP90 protein in 4T1 cells after various treatments (Scale bar: 20 μm). (**F**) CLSM images of the expression of the HSP70 protein in 4T1 cells after various treatments (Scale bar: 20 μm). (* *p* < 0.05, ** *p* < 0.01, *** *p* < 0.001).

The cytotoxicity of various formulations was evaluated in 4T1 cells. As shown in Figure 3A, PD/GA-LPs-NEs exhibited higher cytotoxicity toward 4T1 cells compared to free GA, which was attributed to the enhanced intracellular uptake of PD/GA-LPs by tumor cells. Furthermore, when exposed to NIR laser irradiation, PD/GA-LPs-NEs demonstrated significantly increased cytotoxicity against 4T1 cells. These results suggested that the photothermal agent PD in PD/GA-LPs was effectively activated by *F. nucleatum*, thereby potentiating its anti-proliferative efficacy against tumors (Figure 3B).

To evaluate the apoptotic and cytotoxic effects of PD/GA-LPs-NEs, 4T1 cells were co-stained with Annexin V-FITC/PI and Calcein AM/PI. As displayed in Figure 3C, the *F. nucleatum*-preprocessed PD/GA-LPs-NEs revealed significantly enhanced cytotoxicity. Notably, PD/GA-LPs-NEs combined with NIR laser irradiation induced near-complete death of 4T1 cells, whereas PD/GA-LPs-NEs alone exhibited minimal cytotoxicity, with only a small fraction of cells undergoing apoptosis. Additionally, PBS-treated controls and laser irradiation alone showed no significant cytostatic effects on 4T1 cell viability.

The cytotoxic efficacy of PD/GA-LPs-NEs was further quantified by an Annexin V-FITC/PI assay. As shown in Figure 3D and Figure S14, only approximately 16.33% of cells in the laser irradiation alone group underwent apoptosis. In contrast, PD/GA-LPs-NEs induced apoptosis in a higher percentage of cells (approximately 25.14%), which was attributed to their enhanced cellular uptake and GA-mediated cytotoxic effects. Notably, when combined with near-infrared (NIR) laser irradiation, the apoptotic rate in PD/GA-LPs-NEs-treated cells was markedly elevated to 41.52%, demonstrating a synergistic tumor-killing effect. These results collectively confirm the superior cytotoxicity of PD/GA-LPs-NEs under laser irradiation, providing a solid rationale for their potential application in tumor therapy.

### 3.5. In Vitro Antitumor Mechanism

Immunofluorescence staining was performed to evaluate GA-mediated suppression of HSP90 and HSP70, which are pathologically overexpressed in tumor cells under PTT-induced hyperthermia. As shown in Figure 3E,F, cells treated with hyperthermia alone (PD-LPs-NEs + NIR) exhibited significantly elevated expression of both HSP90 and HSP70 compared to untreated cells. However, the expression levels of HSP90 and HSP70 were effectively suppressed by free GA, suggesting the inhibitory capacity of GA on HSP90 and HSP70 [39]. Notably, a significant downregulation of both HSP90 and HSP70 was observed in 4T1 cells treated with PD/GA-LPs-NEs, regardless of laser irradiation, compared to the PBS control group. These results collectively illustrated that the combination of PD/GA-LPs-NEs and NIR laser irradiation effectively counteracted tumor thermotolerance, thereby potentiating photothermal tumor ablation.

### 3.6. Intracellular ROS Generation

**Figure 4:**
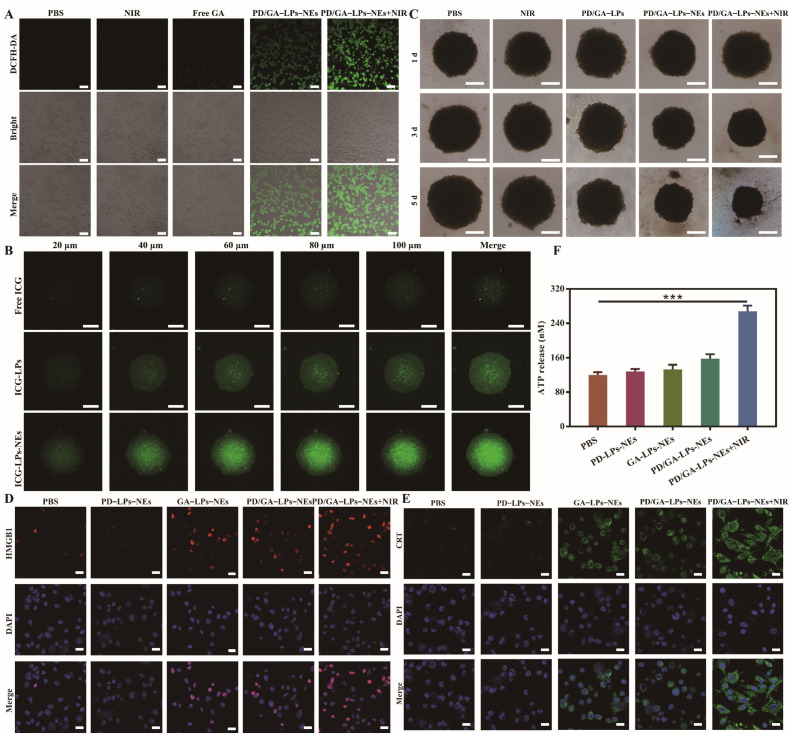
The inhibitory effect of PD/GA–LPs on tumor spheroid proliferation and the PTT–induced ICD in vitro. (**A**) ROS production in 4T1 cells treated with PBS, free GA, PD/GA–LPs-NEs with or without irradiation. (**B**) CLSM images of the in vitro penetration of ICG-LPs-NEs into the 3D tumor spheroids (Scale bar: 100 μm). (**C**) Cell viability of 3D tumor spheroids after incubation with different formulations for 5 d (Scale bar: 200 μm). (**D**) CLSM images of exposed HMGB1 in 4T1 cells with various formulations as indicated (Scale bar: 20 μm). (**E**) CLSM images of exposed CRT in 4T1 cells with various formulations as indicated (Scale bar: 20 μm). (**F**) ATP secretion in 4T1 cells after treatment with various formulations (n = 3) (*** *p* < 0.001).

To investigate the effect of PD/GA-LPs-NEs on intracellular ROS generation in 4T1 cells, DCFH-DA was used to determine the intracellular ROS levels following treatment with free GA or PD/GA-LPs-NEs. As exhibited in Figure 4A, the cells treated with PBS or laser irradiation alone displayed negligible fluorescence signals, suggesting that the intrinsic ROS levels in the tumor cells were relatively low. In contrast, both free GA and PD/GA-LPs-NEs induced a marked increase in green fluorescence intensity, demonstrating significant ROS accumulation. Notably, PD/GA-LPs-NEs combined with NIR laser irradiation triggered the highest ROS production levels in 4T1 cells. These results collectively demonstrate that PD/GA-LPs-NEs synergistically enhance ROS generation under laser irradiation, thereby promoting tumor cell apoptosis.

### 3.7. Tumor Permeability and Cytotoxicity in 4T1 Tumor Spheroids

The aforementioned findings have demonstrated the potential of PD/GA-LPs-NEs as an effective formulation with encouraging anti-tumor activity in 2D cell culture platforms. Nevertheless, the 2D cell culture analysis was unable to predict whether the NEs increased PD/GA-LPs penetration in 3D tumors. Taking this into consideration, we constructed 4T1 tumor spheroids and employed them as an alternative in vitro assessment system to examine the penetration and therapeutic effects of PD/GA-LPs-NEs. As shown in Figure 4B, ICG-LPs-NEs demonstrated superior penetration capabilities at all scanning depths compared with ICG-LPs and free GA, indicating that NEs enhanced the diffusion rate of ICG-LPs in tumor spheroids and promoted intracellular uptake. The tumor spheroid inhibition assay revealed a substantial reduction in tumor spheroid size after treatment with PD/GA-LPs-NEs, especially when combined with 808 nm laser irradiation (Figure 4C and Figure S15). These results provided preliminary evidence that PD/GA-LPs-NEs could be employed to inhibit tumor proliferation, which was a prerequisite for the success of in vivo tumor treatment.

### 3.8. PTT-Induced ICD In Vitro

The PTT-induced ICD response in tumor cells effectively achieves cytotoxicity and triggers antitumor immune enhancement. ICD is characterized by the surface exposure of calreticulin (CRT) on tumor cells, along with the passive release of ATP and high-mobility group box 1 protein (HMGB1). These characteristics enhance dendritic cell-mediated tumor antigen phagocytosis and presentation, thereby potentiating cytotoxic T lymphocyte activation and differentiation. Immunofluorescence analysis revealed that the laser irradiation of 4T1 cells pre-incubated with PD/GA-LPs-NEs triggered intense HMGB1 (red) and CRT (green) fluorescence signals, demonstrating significantly enhanced cytoplasmic translocation of these damage-associated molecular patterns, compared to the modest induction observed in the GA-LPs-NEs and PD/GA-LPs-NEs groups (Figure 4D,E). However, the fluorescence signals were almost undetectable in PBS and PD-LPs-NEs groups. Moreover, 4T1 cells treated with PD/GA-LPs-NEs under NIR irradiation demonstrated a 2.2-fold increase in extracellular ATP release, compared with the PBS group. In conclusion, PD/GA-LPs-NEs-mediated PTT induces tumor ICD, consequently promoting DCs’ maturation, thereby activating a potent anti-tumor immune response.

### 3.9. In Vivo Biodistribution and Photothermal Efficacy

**Figure 5:**
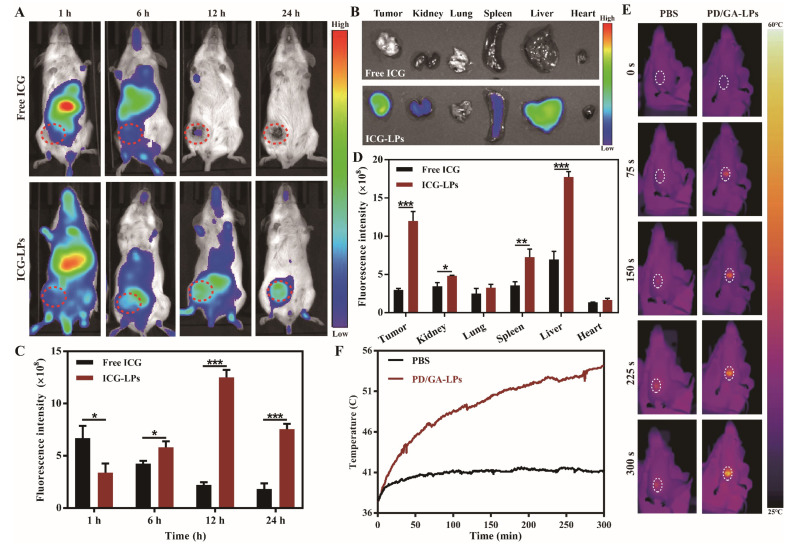
In vivo biodistribution and photothermal profile of PD/GA–LPs in 4T1 tumor-bearing mice. (**A**) In vivo fluorescence images of 4T1 tumor-bearing mice captured at the indicated time points after intravenous administration of free ICG and ICG–LPs. (**B**) The quantification of the relative fluorescence signals of free ICG and ICG-LPs in the tumor site at different time points. (**C**) Ex vivo fluorescence images of major organs excised from mice intravenously injected with free ICG and ICG–LPs at 24 h of postinjection (n = 3). (**D**) Quantification analysis of relative fluorescence intensity in the organs at 24 h after intravenous injection (n = 3). (**E**) Infrared thermographic images of tumors of mice injected with PBS and PD/GA–LPs with laser irradiation at 24 h after injection. (**F**) Time-dependent tumor temperature increase of 4T1 tumor-bearing mice irradiated by the 808 nm laser at 24 h after i.v. injection of PBS and PD/GA–LPs (n = 3) (* *p* < 0.05, ** *p* < 0.01, *** *p* < 0.001).

Following these promising in vitro results, we evaluated the tumor targeting efficiency of LPs in vivo. ICG-LPs were injected intravenously into 4T1 tumor-bearing mice, and the fluorescence intensity of tumors was measured at various time points utilizing real-time fluorescence imaging technology. As shown in Figure 5A, free ICG was distributed mainly to the liver and was followed by rapid clearance from the body. In contrast, ICG-LPs maintained a significant fluorescence signal in the tumor at 24 h post-injection, which was attributed to the tumor-homing capability of NEs mediated by chemotactic factors (Figure 5B) [42]. The in vitro fluorescent images of major organs and tumors had a similar tendency to that presented above at 24 h post-injection (Figure 5C,D). Subsequently, the photothermal effect of PD/GA-LPs on 4T1 tumor-bearing mice was investigated under NIR irradiation (808 nm, 2 W/cm^2^). As anticipated, Figure 5E,F indicates that the temperature at the tumor location rose sharply initially and then gradually increased as the irradiation period prolonged. The temperature of the tumor site in the PD/GA-LPs group reached approximately 54.2 °C, whereas the tumor surface temperature of mice treated with PBS reached only 41.2 °C. These results demonstrated that PD/GA-LPs achieved enhanced accumulation at the tumor site and mediated effective photothermal therapy via *F. nucleatum*.

### 3.10. In Vivo Anti-Tumor Efficiency

**Figure 6:**
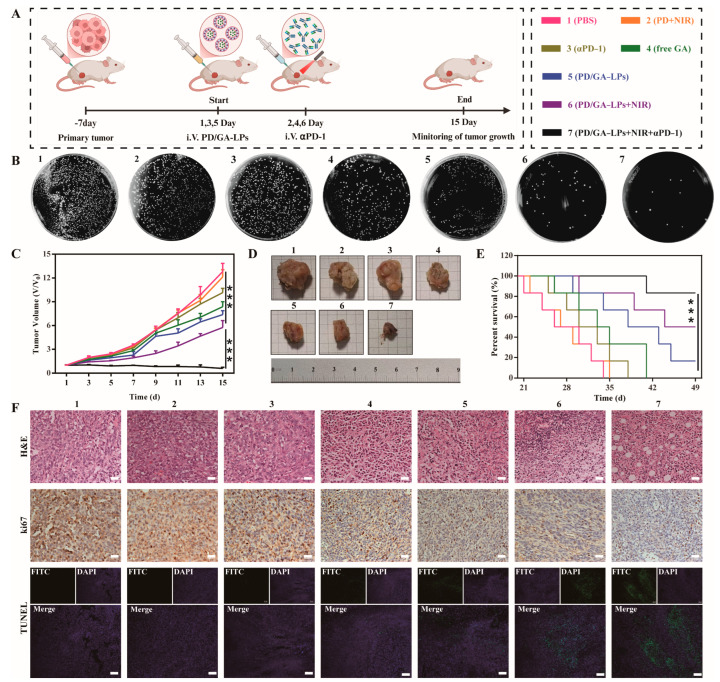
In vivo antitumor evaluation. (**A**) Schematic illustration of the procedure of administration in vivo. (**B**) Images of tumors harvested from 4T1-bearing mice on day 15 after various treatments. (**C**) The growth profiles of 4T1 tumors in mice that received different treatments. (**D**) Survival curves of 4T1 tumor-bearing mice after various treatments. (**E**) Colony plate images of tumors harvested from mice receiving different treatments on their 15th day. (**F**) H&E staining, Ki-67, and TUNEL immunohistochemical images of the tumor harvested from 4T1-bearing mice after different treatments (Scale bar: 100 µm) (*** *p* < 0.001).

The in vivo anti-tumor effect of PD/GA-LPs was investigated in a 4T1 orthotopic tumor model. Tumor-bearing mice were randomly divided into eight groups and treated with one of the following formulations according to the timeline (Figure 6A): (1) PBS, (2) PD + NIR, (3) αPD-1, (4) free GA, (5) PD/GA-LPs, (6) PD/GA-LPs + NIR, and (7) PD/GA-LPs + NIR + αPD-1. To assess the capability of intratumoral bacterial suppression, tumors were excised from the mice, homogenized, and cultured on BHI agar plates on day 15. The number of colonized bacterial colonies was determined through colony enumeration. As depicted in Figure 6B, the PBS, PD + NIR, and αPD-1 groups exhibited minimal impact on the suppression of intratumoral bacteria, while the other groups showed significant antibacterial capabilities. Notably, the PD/GA-LPs + NIR group exhibited the strongest antibacterial activity against intratumoral *F. nucleatum*, with an antibacterial rate of 84.6%, which was 1.22-fold higher than that of the PD/GA-LPs group and 1.82 times higher than that of the free GA group (Figure S16). These results suggest that the combined use of PD/GA-LPs and the photothermal effect could effectively enhance the synergistic inhibitory effect on intratumoral bacteria. Figure 6C illustrates the change in the tumor volume during and after treatment. The tumors in the various formulations grew at a slower rate compared to the PBS group, suggesting that these formulations possessed a general inhibitory effect. Among them, the tumor volume of mice treated with PD/GA-LPs + NIR + αPD-1 declined steadily as the number of days increased, nearly vanishing after 15 days (Figure S17). This result demonstrates the remarkable anti-tumor efficacy of PD/GA-LPs combined with NIR and αPD-1, which almost completely eradicated the primary tumor in situ (Figure 6D). Consistent with the tumor growth results, 83.3% of mice receiving PD/GA-LPs + NIR + αPD-1 survived at least 49 days, whereas none of the mice treated with PBS or PD + NIR survived beyond 35 days (Figure 6E). In addition, as shown in Figure 6F, the most severe tissue damage and minimal proliferation were observed in the tumor tissue of PD/GA-LPs + NIR + αPD-1 group, indicating that it had the strongest antitumor effect among the formulations.

### 3.11. Immune Activation and Antimetastatic Effect In Vivo

**Figure 7:**
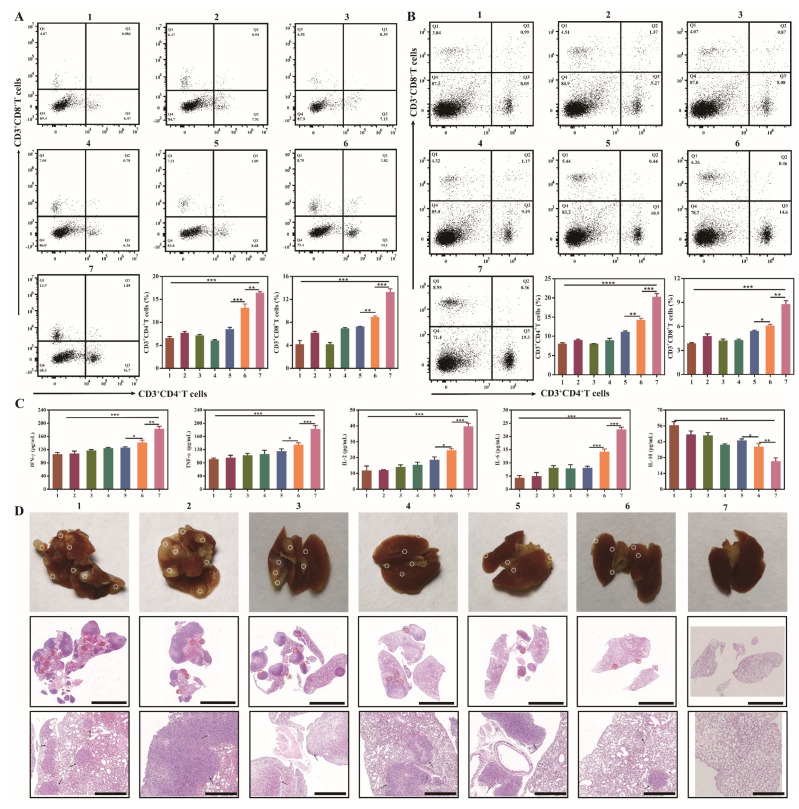
Immune activation and antimetastatic effect of the PD/GA-LPs in vivo. (**A**) Flow cytometry analysis and corresponding quantitative analysis of cytotoxic T lymphocytes gated at CD3^+^CD4^+^ CD8^+^ T cells from primary tumors (n = 3). (**B**) Flow cytometry analysis and corresponding quantitative analysis of cytotoxic T lymphocytes gated at CD3^+^CD4^+^ CD8^+^ cells from spleens (n = 3). (**C**) IFN-γ, TNF-α, IL-2, IL-6, and IL-10 cytokine levels in serum isolated from treated mice (n = 3). (**D**) Representative photographs and H&E staining of lungs in each group (Scale bar: 5 μm, 1 μm) (* *p* < 0.05, ** *p* < 0.01, *** *p* < 0.001, *** *p* < 0.0001).

The combination of photothermal and immunological therapy confirmed that PD/GA-LPs exhibited excellent therapeutic efficacy by killing both tumor cells and tumor-resident bacteria. Moreover, PTT-induced tumor cell death and intratumoral bacterial elimination released substantial amounts of cellular debris and lipopolysaccharides, triggering robust cellular immune responses that effectively inhibit tumor metastasis. Also, given the significant ICD-eliciting capacity observed in cellular models, the in vivo immune responses were evaluated next. The infiltration of CD4^+^ and CD8^+^ T cells in tumors and spleen was determined by flow cytometry analysis. Figure 7A showed that the percentages of CD4^+^ and CD8^+^ T cells in tumors of the PBS group were 6.47% and 4.07%, while the treatment with the PD/GA-LPs + NIR group increased the percentages to 14.1% and 8.75%, respectively. Moreover, αPD-1 antibody treatment further enhanced CD4^+^ and CD8^+^ T cell activation, which increased the percentages to 16.7% and 13.9%, respectively, indicating enhanced infiltration of T cells. Interestingly, flow cytometric analysis revealed stable CD8^+^ T cell infiltration and only nominal variation in CD4^+^ populations, implying a potential link between *F. nucleatum* colonization and PD-1 checkpoint blockade resistance [43]. Emerging evidence in breast cancer research has demonstrated that *F. nucleatum*-derived succinate induces tumor resistance to immune checkpoint inhibitors, including anti-PD-1 antibodies, through modulation of the tumor-immune microenvironment. Moreover, the existence of *F. nucleatum* could reduce the number of CD4^+^ and CD8^+^ T cells in the tumor microenvironment by inhibiting the secretion of CCL5 and CXCL10 from tumor cells, leading to a diminished response to immunotherapy [16,44]. Consistent with the tumor site flow cytometry results, increased CD4^+^ T and CD8^+^ T cell populations were observed in the spleen (Figure 7B). Immune cytokine assays demonstrated that the PD/GA-LPs + NIR + αPD-1 significantly elevated pro-inflammatory mediators (TNF-α, IFN-γ, IL-6, IL-2) while suppressing immunosuppressive IL-10 (Figure 7C). These results indicated that the combination of PD/GA-LPs + NIR and αPD-1 reshapes the tumor microenvironment, lowering the immune suppressive populations and increasing the number of activated T cells.

The immune-memory effect is a hallmark of adaptive immune responses, crucial for the prevention of tumor recurrence and metastasis. To ascertain the potential of combined PTT-immunotherapy in inducing an immune-memory effect, mice with 4T1 tumors were initially subjected to the specified treatments. Subsequently, these mice were intravenously injected with 4T1 cells to simulate cancer cell dissemination. As evidenced by the photographs and H&E staining images of the excised lungs, macroscopic lung metastases were observed in the groups treated with PBS, PD + NIR, αPD-1, GA, PD/GA-LPs, and PD/GA-LPs + NIR. In contrast, the combined treatment of PD/GA-LPs + NIR and αPD-1 effectively inhibited the lung colonization of invasive 4T1 cells, thereby demonstrating the superior immune-memory and protective effects elicited by PD/GA-LP-mediated PTT in conjunction with the PD-1 blockade (Figure 7D).

### 3.12. In Vitro and In Vivo Safety Evaluation

**Figure 8:**
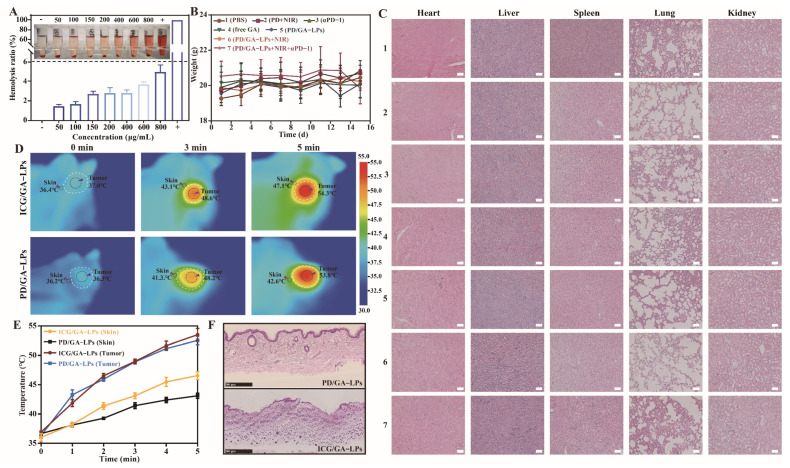
The biosafety evaluation of PD/GA-LPs. (**A**) Hemolysis ratios and hemolysis images of PD/GA-LPs (n = 3). (**B**) H&E staining of vital organ sections after various treatments on 4T1 tumor-bearing. (**C**) Body weight of 4T1 tumor-bearing mice after being treated with various formulations ale bar: 100 μm). (**D**) Infrared thermal images and the temperature evolution of the tumor and skin tissue of mice treated with ICG/GA-LPs and PD/GA-LPs at 808 nm laser irradiation. (**E**) Time-dependent tumor temperature increase of 4T1 tumor-bearing mice irradiated by the 808 nm laser (n = 3). (**F**) H&E section of the skin of 4T1 tumor-bearing mice irradiated by the 808 nm laser (Scale bar: 500 μm).

The biocompatibility of PD/GA-LPs was evaluated through hemolysis detection, body weight monitoring, skin irritation assessment, and H&E staining of major organs in PD/GA-LPs-treated mice. As shown in Figure 8A, PD/GA-LPs demonstrated good hemocompatibility with a hemolysis rate below 6%, validating their blood safety for in vivo applications. Additionally, no significant body weight loss was observed in any group of mice during the 15-day treatment period (Figure 8B). H&E staining results further indicated low systemic toxicity and the minimal side effects of PD/GA-LPs (Figure 8C), which aligned with the blood biochemical indicators (Figure S18). These findings collectively confirmed the biosafety of the prepared PD/GA-LPs.

The ultimate goal of photothermal therapy is to induce targeted apoptosis in tumor tissue while sparing healthy adjacent tissues from thermal injury. By leveraging the distinct biological/microenvironmental differences between tumor and healthy tissues, PD/GA-LPs demonstrated tumor-targeting selectivity, enabling microenvironment-responsive photothermal ablation at tumor sites. Building on these findings, a single-spot experimental model was established to evaluate laser-induced dermal damage caused by photothermal agents. This model comparatively analyzed the temperature differentials between tumor sites and adjacent cutaneous tissues, along with thermal injury manifestations for both ICG/GA-LPs and PD/GA-LPs formulations under identical laser parameters. As demonstrated in Figure 8D,E, the PD/GA-LPs group generated a significantly higher tumor-to-skin temperature gradient (8–12 °C) than ICG/GA-LPs (4–6 °C). This stark contrast underscores PD/GA-LPs’ tumor-selective photothermal targeting, whereas the lower gradient in ICG/GA-LPs failed to confine thermal effects to the tumor site. The insufficient thermal specificity of ICG/GA-LPs resulted in pronounced off-target effects, while PD/GA-LPs exhibited reduced thermal damage to surrounding normal tissues under identical irradiation parameters, demonstrating superior thermal-targeting specificity. H&E-stained post-treatment tissues (Figure 8F) further corroborated these findings, revealing more severe thermal lesions in ICG/GA-LPs-treated skin compared to the PD/GA-LPs group. Collectively, these results validate PD/GA-LPs’ ability to enhance tumor-specific photothermal ablation while effectively sparing peritumoral healthy tissues.

## 4. Conclusions

In summary, we developed a neutrophil-camouflaged stealth liposome (PD/GA-LP) to transform intratumoral bacteria and tumor cells into immunostimulants for BC immunotherapy. Leveraging inflammation-driven chemotactic interactions at the tumor site, the PD/GA-LPs achieved efficient tumor accumulation via neutrophil-assisted delivery. PD released from PD/GA-LPs could be activated by intratumoral bacteria to exert tumor-selective photothermal effects upon bacterial activation. Upon combined treatment with GA, this liposome resulted in potent suppression of both intratumoral bacterial colonization and tumor cell proliferation, triggered robust immunogenic cell death, and substantially enhanced immunogenicity. More importantly, the dead *F. nucleatum* as a potentiator further facilitated the maturation of dendritic cells and subsequent T-cell infiltration. Taken together, the liposomes not only counteracted the therapeutic impediments posed by *F. nucleatum* in cancer treatment but also effectively reprogrammed the TIME, thereby potentiating antitumor immune responses to their maximal capacity. Concurrently, this delivery system also exhibited tumor-selective PTT functionality, which spatially confined thermal effects to neoplastic regions, effectively mitigating off-target heat damage to adjacent normal tissues. Compared to other photothermal-immunotherapy combinations, this strategy not only provides new insights into the development of precise and effective immunotherapy for BC but also significantly enhances the safety of photothermal therapy based on the unique microecology of the tumor. In addition, this strategy may also provide an applicable strategy for the eradication of other intratumoral bacteria, such as centerotoxigenic Bacteroides fragilis and Escherichia coli. Yang and Wang et al. confirmed that perylene diimide derivatives could be selectively reduced to radical anions in situ by Escherichia coli, ultimately exerting selective photothermal therapeutic effects on the bacteria [22,23]. While this preclinical investigation provides foundational insights, several inherent limitations warrant systematic elucidation. The selected murine BC models only partially recapitulated the clinical heterogeneity of human BC, particularly in terms of tumor microenvironment complexity, hormonal receptor expression patterns, and metastatic organotropism. Moreover, fundamental interspecies disparities, including immunological divergence and pharmacological variations, complicate the direct extrapolation of preclinical results to clinical contexts. Overall, this strategy, which is based on the distinction of microecology between tumor and normal tissue and the microbiome-induced reversal of the immune suppression microenvironment, opens new opportunities for precise and efficient immune treatment of BC.

## Data Availability

The data presented in this study are available on request from the corresponding author. The data are not publicly available due to privacy.

## Supplementary Materials

The following supporting information can be downloaded at https://www.mdpi.com/article/10.3390/pharmaceutics17050614/s1: Figure S1: Chemical structures of PD and PD radical anion; Figure S2: UV–vis spectra and fluorescence spectra of PD with varying molar ratios of PDD to CB[7]. (**A**) UV–vis spectra. (**B**) Fluorescence spectra; Figure S3: The stability of PD/GA-LPs in PBS (pH7.4) at 25 °C for 9 d (n = 3). (**A**) Size and polydispersity index. (**B**) Zeta potential; Figure S4: The representative image of PD/GA-LPs before and after incubation with *F. nucleatum*; Figure S5: UV–vis spectra of PD/GA-LPs before and after incubation with *F. nucleatum*; Figure S6: Photothermal stability of PD/GA-LPs upon 808 nm laser irradiation (2W/cm2); Figure S7: Flow cytometry analysis of neutrophil purity; Figure S8: Flow cytometry analysis of DiI-LPs after incubation with NEs for 4 h (n = 3). (*** *p* < 0.001); Figure S9: Representative confocal immunofluorescence microscopy images of neutrophils isolated from peripheral blood 0.5 h after injection of DiI-LPs and stained with anti-CD11b (green) and DAPI (blue). Figure S10: Morphological images of NEs and PD/GA-LPs-NEs stained with SA-β-Gal and DAPI. (Scale bar: 10 μm, 20 μm); Figure S11: The cytotoxicity of PD/GA-LPs after incubation with neutrophils for 24 h at different concentrations (GA: 0.5–50 μg/mL) (n = 4); Figure S12: The representative images of NEs transported in the lower chamber of the Transwell system in the presence of fMLP. (Scale bar: 50 μm); Figure S13: TEM images of PD/GA-LPs released from NEs following treatment with PMA for 4 h. (Scale bar: 300 μm); Figure S14: Quantitative analysis of the apoptosis rate. (** *p* < 0.01, *** *p* < 0.001); Figure S15: Quantitative analysis of 3D tumor spheroids after incubation with different formulations for 5 d. (n = 3); Figure S16: Survival rate of bacteria in the tumor after treatment with different formulations. (n = 3) (1: PBS, 2: PD + NIR, 3: αPD-1, 4: free GA, 5: PD/GA-LPs, 6: PD/GA-LPs + NIR, 7: PD/GA-LPs + NIR + αPD-1); Figure S17: The representative images of mice in each group after treatment with different formulations. (1: PBS, 2: PD + NIR, 3: αPD-1, 4: free GA, 5: PD/GA-LPs, 6: PD/GA-LPs + NIR, 7: PD/GA-LPs + NIR + αPD-1); Figure S18: The blood routine analysis of PD/GA-LPs after treatment with different formulations. (1: PBS, 2: PD + NIR, 3: αPD-1, 4: free GA, 5: PD/GA-LPs, 6: PD/GA-LPs + NIR, 7: PD/GA-LPs + NIR + αPD-1).

## Abbreviations

BC	Breast cancer
BHI	Brain heart infusion
CLSM	Confocal laser scanning microscopy
CRT	Calreticulin
DAMPs	Damage-associated molecular patterns
DL	Drug loading
DLS	Dynamic light scattering
DOCP	Inverse phosphocholine lipids
EE	Encapsulation efficiency
GA	Gambogic acid
HMGB1	High-mobility group box 1 protein
ICD	Immunogenic cell death
ICIs	Immune checkpoint inhibitors
ICG	Indocyanine green
NEs	Neutrophiles
NETs	Neutrophile extracellular traps
NIR	Near infrared laser
PAMPs	Pathogen-associated molecular patterns
PBS	Phosphate buffer solution
PDD	Perylene diimide derivatives
PDI	Polydispersity index
PD-1	Programmed cell death protein 1
PMA	Phorbol 12-myristate 13-acetate
PTT	Photothermal therapy
SEM	Scanning electron microscopy
TEM	Transmission electron microscopy
