# Correction: Chen et al. Neutrophil-Camouflaged Stealth Liposomes for Photothermal-Induced Tumor Immunotherapy Through Intratumoral Bacterial Activation. *Pharmaceutics* 2025, *17*, 614

**DOI:** 10.3390/pharmaceutics17121615

**Published:** 2025-12-16

**Authors:** Xinxin Chen, Jiang Sun, Tingxian Ye, Fanzhu Li

**Affiliations:** 1 School of Pharmaceutical Sciences, Zhejiang Chinese Medical University, Hangzhou 310053, China; m13884490537@163.com (X.C.); yetingxian123@foxmail.com (T.Y.); 2 Jinhua Academy of Zhejiang Chinese Medical University, Jinhua 321015, China; sunj500@163.com

**Error in Figure**

In the original publication [1], there was a mistake in Figures 3 and 6 as published. The authors would like to make the following updates to the published article [1] to ensure the clarity and completeness of the presented data. An error occurred during the editing of Figure 3, where the Calcein images of the PBS group and the PD/GA-LPS-NE group were incorrectly arranged. Upon reviewing the raw data, we confirmed that we completely merged the images for the PD/GA-LPS-NE group. In Figure 6, the photographs for Group 6 and Group 7 were mistakenly imported during the processing of images captured by mobile phones. We also noted that the Ki67 images of Group 4 and Group 5, as well as the TUNEL images of Group 2 and Group 5, were accidentally duplicated due to missing links in the original images in the Adobe Illustrator workflow. Furthermore, immunofluorescence images from the PBS group were used as substitutes to address the erroneous application of TUNEL images in Group 1, resulting from the broken file links during the migration of the original folder. The corrected Figure 3 and Figure 6 are presented below. The authors state that the scientific conclusions are unaffected. This correction was approved by the Academic Editor. The original publication has also been updated.

## Figures and Tables

**Figure 3:**
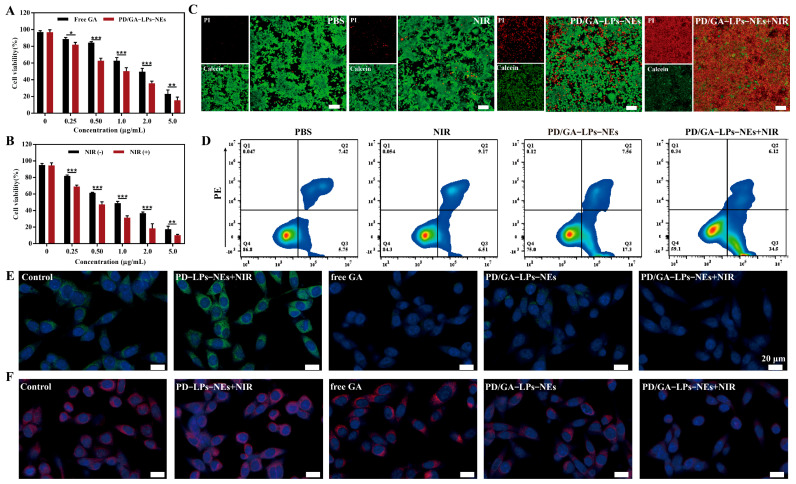
In vitro anticancer effects of PD/GA-LPs against 4T1 cells. (**A**) Cell viability of 4T1 cells incubated with various formulations for 24 h (n = 4). (**B**) Cell viability of 4T1 cells after PD/GA-LPs-NEs treatment for 24 h with or without laser irradiation (n = 4). (**C**) Live-dead staining of 4T1 treated with PBS and different formulations for 4 h (Scale bar: 100 µm). (**D**) Apoptosis analysis via an Annexin V-FITC/PI assay of 4T1 cells incubated with PBS and different formulations for 4 h (n = 3). (**E**) CLSM images of the expression of the HSP90 protein in 4T1 cells after various treatments (Scale bar: 20 µm). (**F**) CLSM images of the expression of the HSP70 protein in 4T1 cells after various treatments (Scale bar: 20 µm). (* *p* < 0.05, ** *p* < 0.01, *** *p* < 0.001).

**Figure 6:**
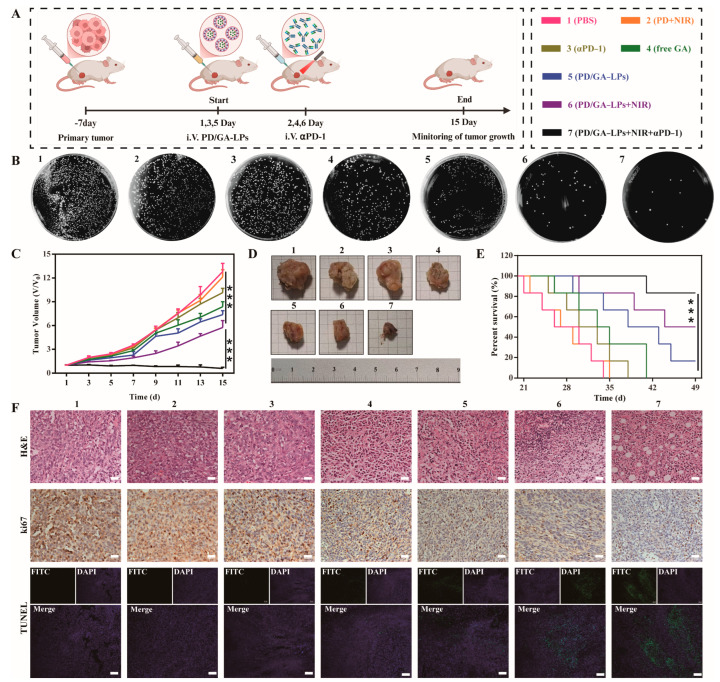
In vivo antitumor evaluation. (**A**) Schematic illustration of the procedure of administration in vivo. (**B**) Images of tumors harvested from 4T1-bearing mice on day 15 after various treatments. (**C**) The growth profiles of 4T1 tumors in mice that received different treatments. (**D**) Survival curves of 4T1 tumor-bearing mice after various treatments. (**E**) Colony plate images of tumors harvested from mice receiving different treatments on their 15th day. (**F**) H&E staining, Ki-67, and TUNEL immunohistochemical images of the tumor harvested from 4T1-bearing mice after different treatments (Scale bar: 100 µm) (*** *p* < 0.001).
