# Supplementary material for: Neutrophil-Camouflaged Stealth Liposomes for Photothermal-Induced Tumor Immunotherapy Through Intratumoral Bacterial Activation
Source: Pharmaceutics. 2025 May 5;17(5):614. doi: 10.3390/pharmaceutics17050614 (PMC12115177; doi:10.3390/pharmaceutics17050614)
Supplement: Supplementary file 1 [file pharmaceutics-17-00614-s001.zip › Supplement materias.pdf]

Supplementary data

Neutrophil camouflaged stealth liposomes for photothermal-induced tumor immunotherapy through intratumoral bacterial activation

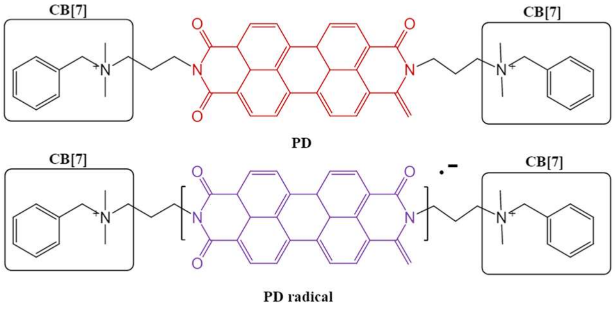

Fig.S1 Chemical structures of PD and PD radical anion.

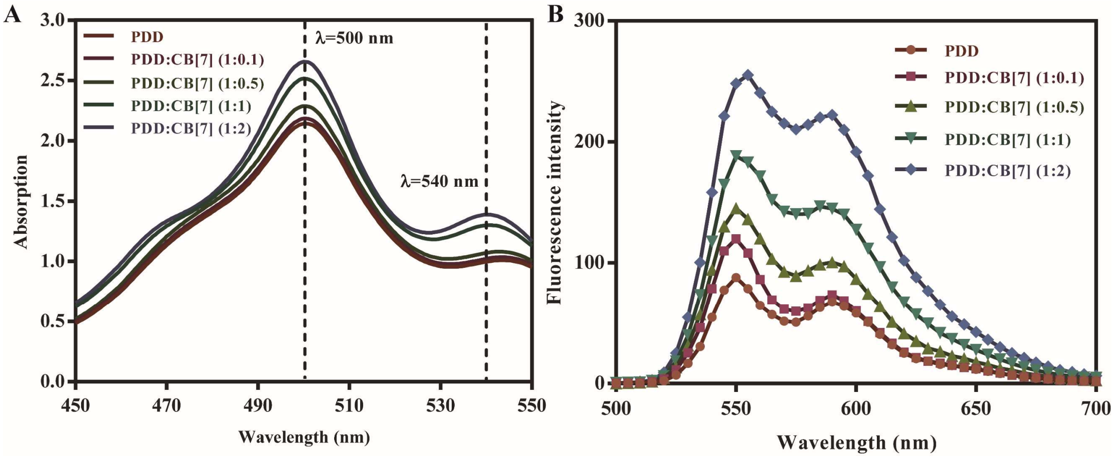

Fig.S2 UV-vis spectra and fluorescence spectra of PD with varying molar ratios of PDD to CB[7]. (A) UV-vis spectra. (B) fluorescence spectra.

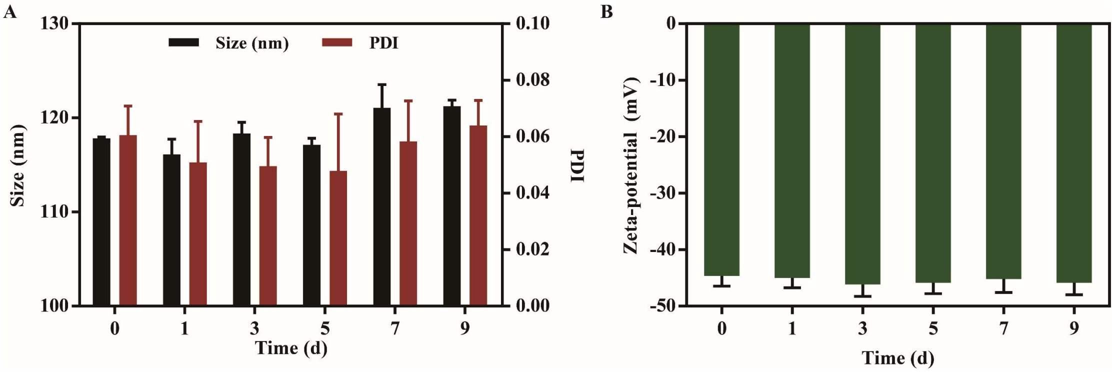

Fig.S3 The stability of PD/GA-LPs in PBS (pH7.4) at 25°C for 9 d (n=3). (A) Size and polydispersity index. (B) Zeta potential.

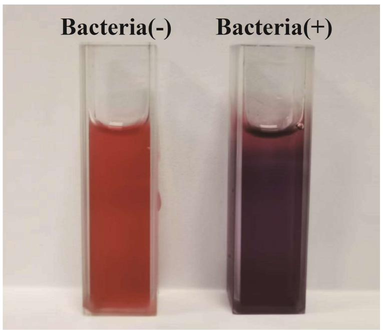

Fig.S4 The representative image of PD/GA-LPs before and after incubation with F. nucleatum.

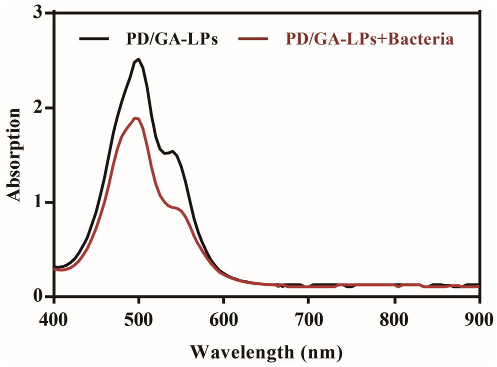

Fig.S5 UV-vis spectra of PD/GA-LPs before and after incubation with F. nucleatum.

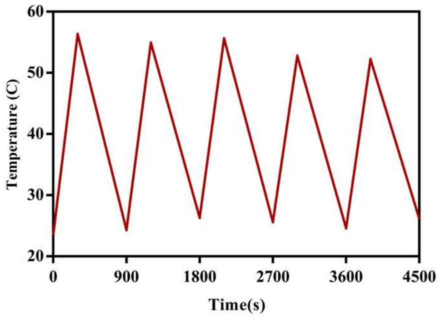

Fig.S6 Photothermal stability of PD/GA-LPs upon 808 nm laser irradiation ($2W/cm^2$).

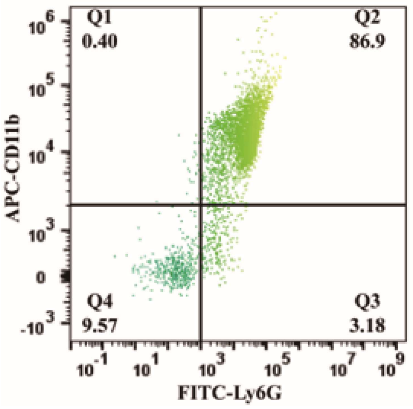

Fig.S7 Flow cytometry analysis of neutrophil purity.

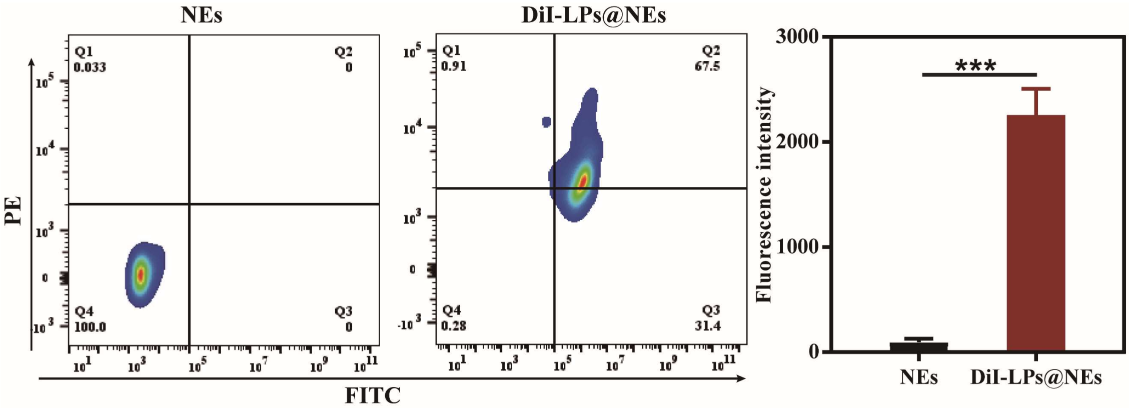

Fig.S8 Flow cytometry analysis of DiI-LPs after incubation with NEs for 4 h (n=3).

(***$P < 0.001$)

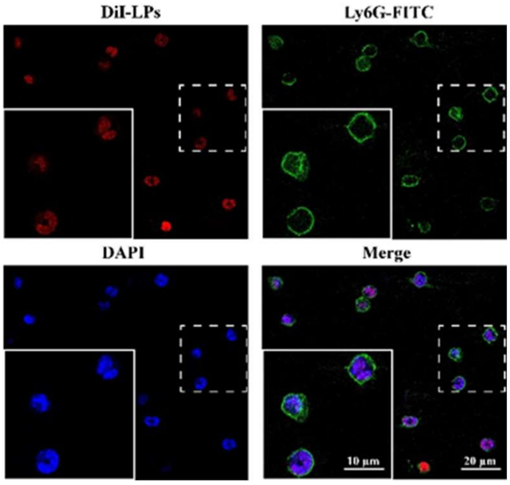

Fig.S9 Representative confocal immunofluorescence microscopy images of neutrophils isolated from peripheral blood 0.5 h after injection of DiI-LPs, and stained with anti-CD11b (green) and DAPI (blue). (Scale bar: 10 μm, 20 μm)

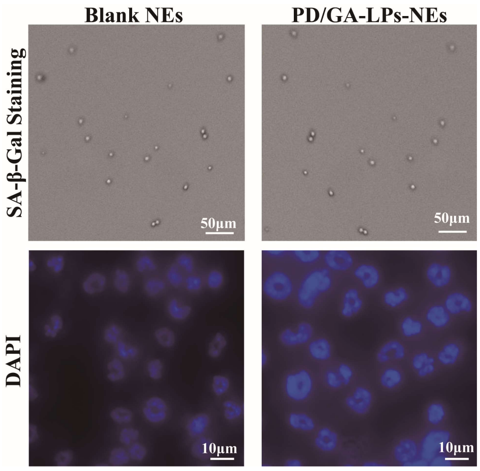

Fig.S10 Morphological images of NEs and PD/GA-LPs-NEs stained with SA-β-Gal

and DAPI. (Scale bar: 10 μm, 50 μm)

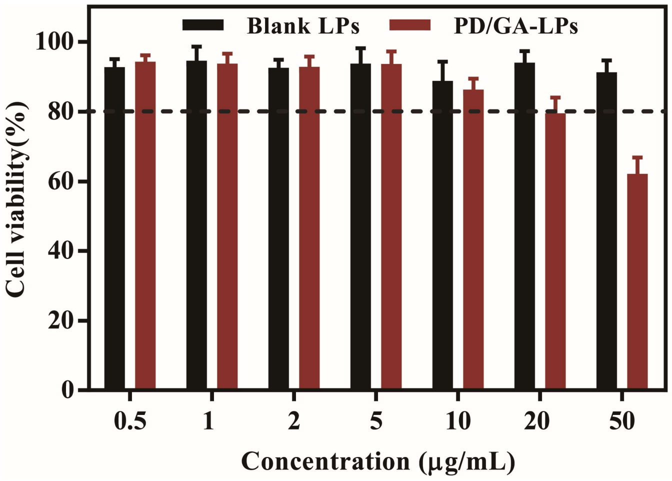

Fig.S11 The cytotoxicity of PD/GA-LPs after incubation with neutrophiles for 24 h at different concentrations (GA: 0.5-50 μg/mL) (n=4).

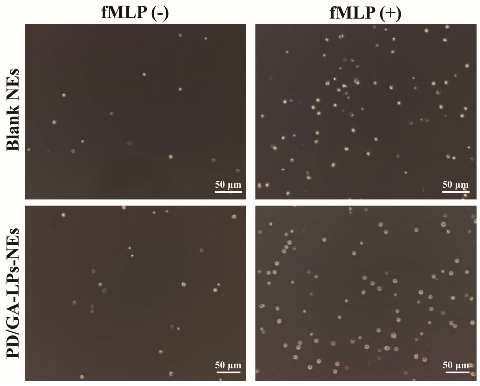

Fig.S12 The representative images of NEs transported in the lower chamber of the Transwell system in the presence of fMLP. (Scale bar: 50 μm)

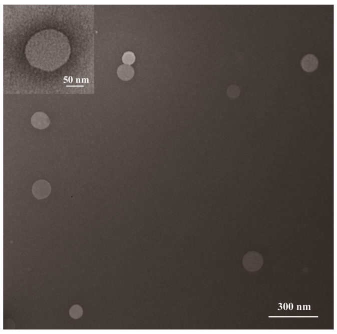

Fig.S13 TEM images of PD/GA-LPs released from NEs following treatment with PMA for 4 h. (Scale bar: 300 μm)

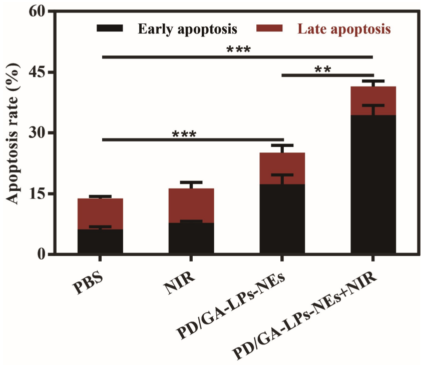

Fig.S14 Quantitative analysis of the apoptosis rate. (**$P < 0.01$, ***$P < 0.001$)

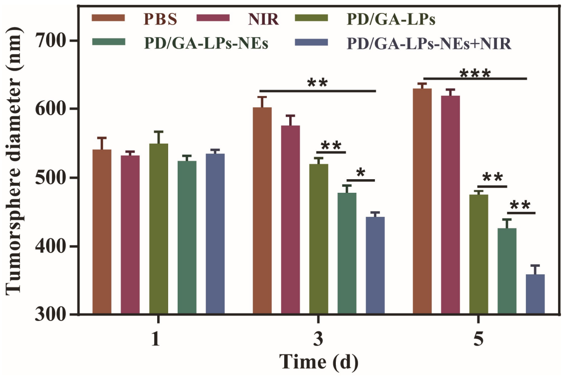

Fig.S15 Quantitative analysis of 3D tumor spheroids after incubation with different formulations for 5 d. (n=3)

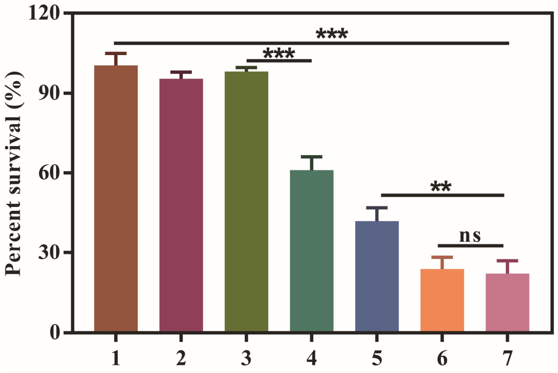

Fig.S16 Survival rate of bacteria in tumor after treatment with different formulations. (n=3) (1: PBS, 2: PD+NIR, 3: αPD-1, 4: free GA, 5: PD/GA-LPs, 6: PD/GA-LPs+NIR, 7: PD/GA-LPs+NIR+αPD-1)

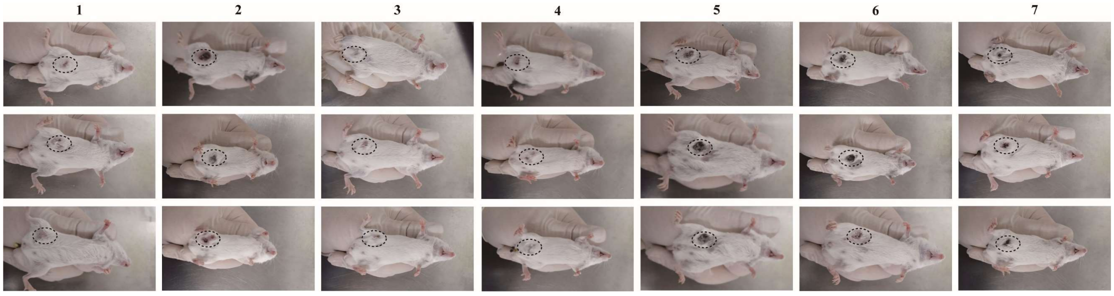

Fig.S17 The representative images of mice in each group after treatment with

different formulations. (1: PBS, 2: PD+NIR, 3: αPD-1, 4: free GA, 5: PD/GA-LPs, 6: PD/GA-LPs+NIR, 7: PD/GA-LPs+NIR+αPD-1)

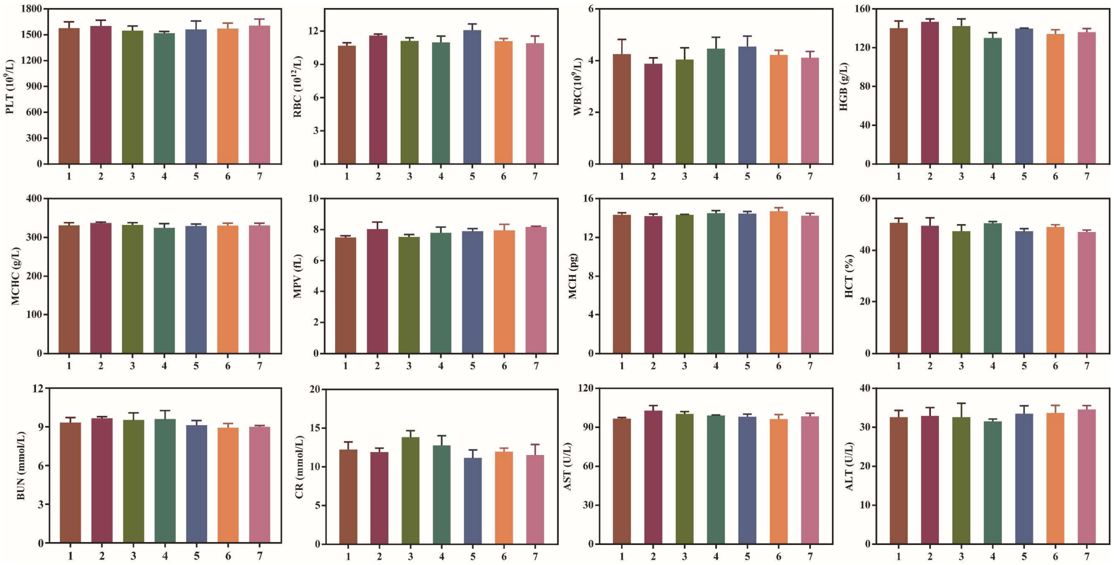

Fig.S18 The blood routine analysis of PD/GA-LPs after treatment with different formulations. (1: PBS, 2: PD+NIR, 3: αPD-1, 4: free GA, 5: PD/GA-LPs, 6: PD/GA-LPs+NIR, 7: PD/GA-LPs+NIR+αPD-1)
